# Asparagine endopeptidase regulates lysosome homeostasis via modulating endomembrane phosphoinositide composition

**DOI:** 10.1038/s41419-024-07187-3

**Published:** 2025-01-02

**Authors:** Linli Yao, GuangHui Zi, Miao He, Yuhong Xu, Lulu Wang, Baowei Peng

**Affiliations:** 1 College of Pharmacy, Dali University, Dali 671003, Yunnan PR China; 2 State Key Laboratory of Oncogenes and Related Genes, Shanghai Cancer Institute, Ren Ji Hospital, School of Medicine, Shanghai Jiao Tong University, Shanghai, PR China; 3 Yunnan Key Laboratory of Screening and Research on Anti-Pathogenic Plant Resources from Western Yunnan, Dali University, Dali 671003, Yunnan PR China; 4 Department of Human Anatomy, School of Basic Medical Sciences, Capital Medical University, Beijing, PR China; 5 Yunnan Provincial Key Laboratory of Entomological Biopharmaceutical R&D, College of Pharmacy, Dali University, Dali 671003, Yunnan PR China

**Keywords:** Cancer, Cell biology, Cancer

## Abstract

Asparagine endopeptidase (AEP) is ubiquitously expressed in both physiological and pathological contexts, yet its precise role and functional mechanism in breast cancer remain elusive. Here, we identified increased AEP expression in breast cancer tissues, which correlated with poorer survival rates and a propensity for lung metastasis among breast cancer patients. Loss of AEP impaired colony formation by breast cancer cells in vitro and suppressed lung metastasis in mice. By Gene Set Enrichment Analysis (GSEA) analysis, we uncovered a positive association between aberrant AEP expression and autophagy as well as lysosomal function. Loss of AEP in breast cancer cells led to reduced autophagosome clearance and impaired lysosomal degradation. Mechanically, by co-immunoprecipitation and in vitro enzymatic cleavage assays, we identified the regulatory subunit p85 of class IA PI3K phosphatidylinositol 3-kinase (PI3K), as a substrate of AEP. Loss of AEP led to elevated endo/lysosomal PI3K activity and subsequent conversion of PtdIns(4,5)P2 (PIP2) to PtdIns(3,4,5)P3 (PIP3) on endo/lysosome membranes. Notably, the novel function of endo/lysosomal PI3K which was differently with its role in cytomembrane, was revealed by pharmacological inhibition with a potent endo/lysosomal PI3K inhibitor PIK75. PIK75 treatment showed increased vacuolar-ATPase assembly endo/lysosome membranes, prevented over lysosome perinuclear clustering/fusion and enhanced autophagosome clearance. Our findings demonstrate that AEP regulates cellular autophagy by modulating lysosomal function through its control over endo/lysosomal PI3K activity. These results suggest that AEP may serve as a potential target for suppressing metabolic adaptations in cancer.

## Highlights

- Increased asparagine endopeptidase (AEP) expression in breast cancer is predictive of poor prognosis in human patients.
- AEP promotes autophagy by regulating lysosome homeostasis in endo/lysosome PI3K-dependent manner.
- P85 of PI3K on lysosome is recognized as a substrate of AEP and degraded by AEP.
- Endo/lysosome PI3K activity regulates endomembrane V-ATPase assembly.

## Introduction

Breast cancer is the leading cause of cancer-related mortality among women globally. The primary factor contributing to breast cancer mortality is the occurrence of distant metastasis to a secondary organ. The survival rate at 5 years for primary breast cancer is almost 99%, but this drops drastically to 23% for those who develop distant metastasis [1, 2]. Autophagy is a vital recycling system that plays a crucial role in enabling cellular adaptation to metabolic stresses [3]. Autophagy has been implicated in cancer metastases by regulating cell survival [4], therefore, elucidating the specific mechanism that regulates autophagy during cancer progression holds significant potential for identifying promising targets for precise antimetastatic treatment in breast cancer.

Autophagy is a dynamic recycling system by which the defective cellular components can be recycled by the cell to meet its metabolic needs. Autophagy plays a key role in the maintenance of cellular homeostasis and renovation under stressful conditions [5]. Intracellular damaged proteins or organelles are encapsulated by autophagosomes, which subsequently fuse with lysosomes to form autolysosomes. The lysosomes are tasked with the digestion of the autophagosome contents, a process that is of utmost importance for the regulation of autophagy and the maintenance of normal cellular metabolic activities. This low pH of 4.5–5.5 in the lysosomal lumen enables the activation of >50 intralysosomal hydrolases, which can effectively digest macromolecules. The acidic lysosomal lumen is maintained by the lysosomal multi-subunit vacuolar-ATPase (V-ATPase). Investigating the molecular regulating of lysosomal function is of great importance for clarifying the cellular metabolic regulation.

Asparagine endopeptidase (AEP) or Legumain (EC 3.4.22.34), is a lysosomal cysteine proteinase that selectively cleaves substrates with an asparagine residue at the P1 site [6, 7]. Mammalian AEP is synthesized as an inactive preproprotein (~56 kDa) and autocatalytically processed to active mature AEP (~36 kDa) at acidic pH [8–11]. AEP has been detected in heart, lung, liver, spleen, testis, thymus and brain tissues, and it is highly abundant in the kidney and placenta [12, 13]. Thus, it is not surprising that AEP has been implicated in a plethora of physiological and pathological conditions [13–20]. In addition, AEP is overexpressed in acute lymphoblastic leukemia [21] and in the majority of human solid tumors [22–29]. AEP-deficient kidney-proximal tubule cells contained greatly enlarged LAMP-2 positive lysosomes [30]. Although the expression of AEP in some tissues is well documented, its function and cell biology in breast cancer, remains to be investigated.

In this study, we identified the metastasis-promoting effect of AEP by enhancing autophagy in breast cancers. Here, we further sought to explore the functional mechanisms of AEP and focused on the potential function of AEP in lysosome function remodeling. We investigated whether AEP regulated endo/lysosomal PI3K activity and its role in lysosome homeostasis and function. Our findings indicate that AEP modulates endo/lysosomal PI3K activity to regulate lysosome homeostasis and degradation, thus affecting cellular metabolism. Our results indicate the AEP-endo/lysosomal PI3K axis may represent a target to suppress metabolic adaptations in breast cancer.

## Material and methods

All methods were performed in accordance with the relevant guidelines and regulations.

### Bioinformatics analysis

In the present study, UCSC XENA dataset containing 1099 samples of BRCA and 113 sample of normal tissue were from The Cancer Genome Atlas (TCGA) and 179 samples of normal tissue were from GTEx were used to evaluate the AEP genes expression in breast cancers. Gene Expression Omnibus (GEO) datasets GSE45255 and GSE GSE25066 were used to evaluate the association between AEP expression and patient survival. ROC analysis was performed in TCGA data and curves were plotted using the function “roc” in the R package. For gene set enrichment analysis (GSEA) of TCGA data, gene expression higher than the median of AEP was designated as “High AEP expression”, while lower than the median was designated as “Low AEP expression”. GSEA was performed on the Broad Institute Platform, and the statistical significance (false discovery rate, FDR) was set at 0.25.

### Cell culture

MDA-MB-231 and 4T1 cells were obtained from ATCC and cultured in Dulbecco’s modified Eagle medium (DMEM) supplemented with 10% fetal bovine serum (FBS), 100 U/ml penicillin, 100 µg/ml streptomycin (complete DMEM). For serum starvation treatment, complete DMEM medium was placed with plain DMEM.

### AEP knockdown

Lentivirus-delivered shRNA was used to knockdown AEP expression in MDA-MB-231 cells as described previously [29].

### siRNA transfection

Breast cancer cell line 231 cells were transfected with siRNAs specific for the catalytic subunits PIK3CA and PIK3CB or regulatory subunits PIK3R1 and PIK3R2 routinely. Cells were harvested 36 h later, lysed and analyzed for the knockdown efficiency of proteins.

### Antibodies and reagents

Antibodies against Legumain (R&D, #AF2199), LC3 (MBL #AM1800a), p62 (Novus #NBP1-48320), cathepsin D (sigma, #C0715), LAMP1 (Sigma, #L1418), Legmumain/AEP (R&D, #AF2199), ATP6V1C1 (#sc-376227), ATP6V0D1 (#sc-393322), ATP6V0A1 (Sigma, #AV46581), phospho-Akt (S473) (CST, #4060), Akt (CST, #4691), phospho-p70S6K (CST, #9234), p70S6K (CST, #2708), mTOR (CST, #2983), Rab5 (CST, #3547), Rab7 (CST, #9367), LAMP2 (Epitomics, #3660-1) were used at 1:1000 dilution as primary antibodies for western blotting. Antibodies against PI(4,5)P2 (Echelon, #Z-G045) and PI(3,4,5)P3 (Echelon, #Z-G345) were used at 1:200 dilution for flow cytometry and immunofluorescence staining. LysoTracker™ Red DND-99 (#L7528), Recombinant human Legumain/AEP (2199-CY-010) were purchased from R&D Systems. Plasmid expressing LAMP1-GFP was obtained from Addgene (#34381). Adenovirus expression mRFP-GFP tandem fluorescence tagged LC3 (#HB-LP2100001) was purchased from Hanbio (Shanghai, China).

### Ratiometric lysosome pH measurement

LysoSensor™ Blue DND-167 (#L7533) purchased from Invitrogen was used to measure lysosome pH as described previously [31].

### Immunofluorescence and confocal microscopy

To detect autophagosome formation, wild-type (WT) and AEP knockdown (AEPKD) MDA-MB-231 cells were transduced with adenovirus expression mRFP-GFP tandem fluorescence tagged LC3 for overnight. Cells were then starved for AAs or FBS, or treated with rapamycin (500 nM), BA1 (100 nM), or PIK75 (100 nM). Autophagosome area were analyzed and quantified by ImageJ. To label late lysosomes, endosomes, cells were pulse-labeled with 2.5 µM Lysotracker, washed, chased and examined by fluorescence microscopy. LAMP2 was detected with primary rabbit anti-LAMP2 antibody (1:400 dilution) and conjugated goat anti-rabbit antibody. LC3 was detected with primary rabbit anti-LC3 and goat anti-rabbit antibody. Usually, cells were blocked with PBS containing 0.1% saponin and 1% normal goat serum or 2% normal donkey serum for 30 min.

### Immunoblotting

Immunoblotting was performed routinely. Briefly, cells were lysed in Triton buffer (20 mM Hepes and 0.5% Triton X-100, pH 7.6). Thirty µg lysates were resolved separated by 10% SDS-PAGE. Proteins were transferred onto polyvinylidene fluoride filters and followed by a blocking step using Tris-buffered saline with 0.1% Tween 20 plus 5% nonfat dried milk for 1 h at room temperature. The filters were then incubated with primary antibody, followed by incubation with secondary antibody conjugated with HRP. After extensive washing of the blots, signals were visualized with chemiluminescent HRP substrate (Millipore). The blot was stripped off and re-probed with other antibodies to detect corresponding proteins.

### Transmission electron microscopy

Cells were fixed in Karnovsky’s fixative (2.5% glutaraldehyde, 4% paraformaldehyde in 100 mM cacodylate buffer pH7.2), then post-fixed in 1%OsO4 in carcodylate buffer for 60 min. The samples were dehydrated in alcohol, transferred to 100% propylene oxide and finally incubated on a rotator in 50% propylene oxide: 50% Durcupan resin. Sections were cut and stained with 3% uranyl acetate followed by lead citrate. Grids were imaged on a Hitachi TEM system.

### In vitro cleavage of GST-p85 by AEP

One µg of GST-p85 was incubated with 1 µg or 2 µg of AEP at 37 °C for 4 h. Reaction was stopped by heating at 95 °C for 10 min and analyzed by Comassie blue staining or immunoblotting with p85 antibody.

### Experimental lung metastasis

Nude mice (female, 6–8 old) were purchased from Slaccas Company (Shanghai, China) and randomly separated to different groups. Single-cell suspension of WT or AEPKD MDA-MB-231 cells (8 × 10^5^/0.1 mL PBS) was injected i.v. into tail vein of each mouse (8 mice/group). At week 4, 3 mice from each group were analyzed. At week eight, rest 5 mice from each group were analyzed. Mice were anaesthetized and subsequently perfused with PBS and PBS-buffered 4% paraformaldehyde. Lungs were dissected and processed for paraffin-embedded sectioning at 5 µm and stained with H&E or with IHC.

Single-cell suspension of WT and AEPKD murine breast cancer cell line 4T1 (5 × 10^5^/0.1 mL were injected i.v. into tail vein of each mouse). Mice were anaesthetized 15 days later. Lungs were examined and metastasis nodules were counted.

All animal studies were approved by the Institutional Animal Care and Use Committee of Dali University (2022-PZ-24).

### Statistical analysis

Data are expressed as mean ± SD, and analyzed by two-tailed and unpaired Student’s *t* test in Excel with significance defined as *p* < 0.05.

## Results

### High AEP expression predicts distant metastasis of BRCA patients and promotes lung metastasis in mice

**Fig. 1:**
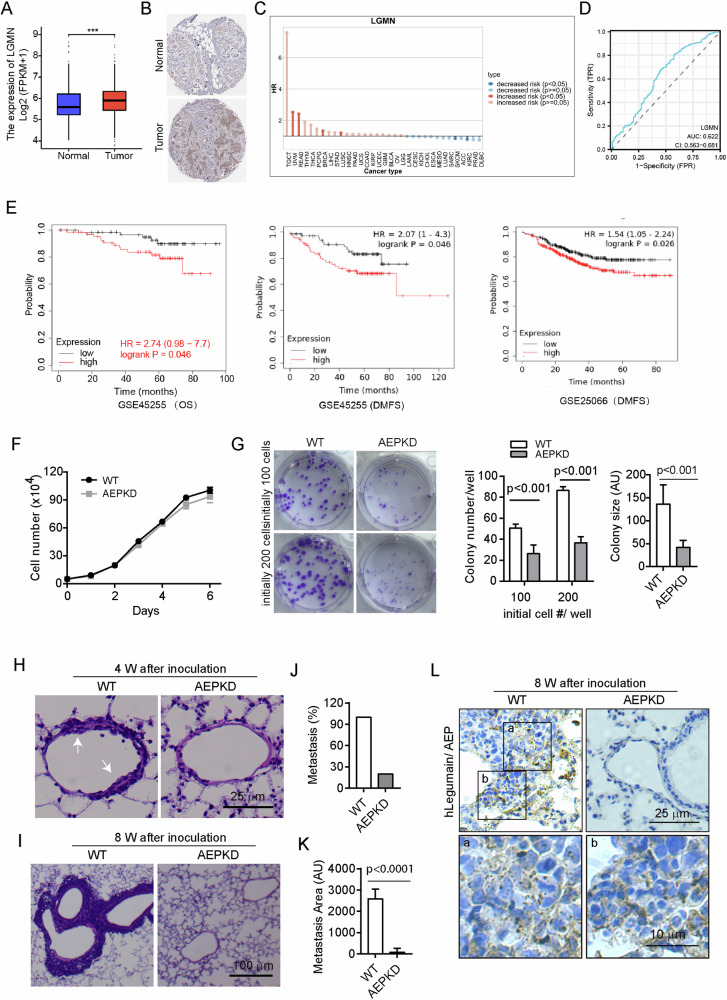
AEP expression is increased in breast cancer and predicts metastasis and poor prognosis. **A** Expression analysis of AEP (coded by LGMN) in tumors (T) and normal (N) breast tissue samples from UCSC XENA dataset. Expression values were compared using Mann-Whitney *U* test: ***P < 0.001 compared with Normal. **B** Representative immunohistochemical images showing AEP expression in BRCA from The Human Protein Atlas (https://www.proteinatlas.org/). **C** Correlation of AEP expression with risk factors in different tumors from TCGA cohort online (TISCH2, http://tisch.comp-genomics.org/home/). **D** ROC curves of AEP using data from TCGA. Samples were obtained from UCSC XENA dataset. **E** Expression of AEP with Overall survival (OS) and Distant Metastasis-Free Survival (DMFS) in two independent Gene Expression Omnibus (GEO) datasets (GSE45255 and GSE25066) of BRCA. **F** Comparison of growth curve of WT and AEPKD MDA-MB-231 cells. Data are mean ± s.d.; *n* = 3. Similar results are observed in three independent experiments. **G** Loss of AEP impairs colony formation by cancer cells in vitro. WT and AEPKD MDA-MB-231 cells were plated at 100 cells/well or 200 cells/well in six-well plate in 2 mL full tissue-culture medium, grown for 14 days without changing medium, and stained with crystal purple solution. The image shown is from representative experiments The right graphs show quantification of colony number/well and colony size. AU arbitrary unit. Data are mean ± s.d.; *n* = 3. Similar results are observed in four independent experiments. **H**-**K** WT and AEPKD MDA-MB-231 cells were inoculated into nude mice via tail vein injection. Mice lungs were dissected and histological sections were made and stained with H&E at indicated times. Apparent hyperplasia (**H**) (*n* = 3 mice/each group) and micro-metastases (**I**) (*n* = 5 mice/each group) around blood vessels were found in lungs of mice inoculated with WT MDA-MB-231 cell. Arrows point to hyperplasia. The images shown are from representative H&E staining. Quantification of percentage of mice with micro-metastasis (**J**) and accumulative metastases area in each group (**K**) at week eight. Data are mean ± s.d.; *n* = 5 mice/each group. AU arbitrary unit. **L** Immunohistochemistry (IHC) detection of human AEP in mice lung metastases at week eight. Histological sections of experimental lung metastasis were stained with antibody specific to human AEP, and visualized with DAB. Outlined regions are enlarged at the bottom row. The images shown are from representative IHC experiments.

To determine the expression changes of AEP (coded by Legumain, LGMN) in BRCA, we analyzed the mRNA levels of AEP in UCSC XENA dataset (1099 samples of BRCA and 113 sample of normal tissue were from The Cancer Genome Atlas (TCGA) and 179 samples of normal tissue were from GTEx). AEP protein expression was shown by Immunohistochemical (IHC) staining from The Human Protein atlas (https://www.proteinatlas.org/; URL for normal image: Tissue expression of LGMN - Staining in breast - The Human Protein Atlas for; URL for tumor image: Expression of LGMN in breast cancer—The Human Protein Atlas) [32–34]. We found both mRNA and protein expression of AEP was increased in breast cancer tumors compared with normal control tissue (Fig. 1A, B). By the analysis online (TISCH2, http://tisch.comp-genomics.org/home/), we found AEP significantly increased the risk in multiple cancers, including Uveal Melanoma (UVM), Rectum adenocarcinoma (READ), Breast invasive carcinoma (BRCA) and Lung squamous cell carcinoma (LUSC) (Fig. 1C). To assess the predictive performance of AEP in BRCA, we performed ROC analysis and used the area under the ROC curve (AUC) as an assessment of the prediction accuracy in TCGA cohort. As shown in Fig. 1D, AEP (AUC = 0.622, CI = 0.563–0.681) had a high accuracy in predicting cancer (Fig. 1D). In two independent Gene Expression Omnibus (GEO) datasets (GSE45255 and GSE25066), we found higher AEP expression was positively associated with shorter Overall survival (OS) and Distant Metastasis-Free Survival (DMFS) (Fig. 1E). These results suggest the tumor-promoting role of AEP in breast cancer patients.

To gain further insight into the oncogenic roles of AEP in BRCA, we performed short hairpin RNA (shRNA)-mediated knockdown of AEP in BRCA cell line MDA-MB-231, which was referred to as AEPKD cells. Loss of AEP did not significantly affect growth of breast cancer cells cultured in bulk in full tissue-culture medium (Fig. 1F), consistent with previous findings that AEP-deficient mice are viable and grow normally at young age [13, 30, 35]. However, loss of AEP significantly impaired the ability of breast cancer cells to form colonies, as shown by less colony number and smaller colony size formed by AEPKD MDA-MB-231 cells compared to that formed by WT MDA-MB-231 cells (Fig. 1G), indicating impaired survival and growth potential of individual AEPKD cancer cell under stressed conditions.

To test the ability of AEPKD cells to survive and metastasis in vivo, we employed experimental lung metastasis for this purpose using both MDA-MB-231 and 4T1 cells. At 4 weeks after i.v. inoculation of MDA-MB-231 single-cell-suspension into nude mice, histological section examination revealed the formation of hyperplasia attached to blood vessels in lungs of mice inoculated with WT MDA-MB-231 cells (3/3) but not AEPKD MDA-MB-231 cells (0/3) (Fig. 1H). At week eight, multiple microscopic metastases were found around blood vessels in all lungs of mice inoculated with WT MDA-MB-231 cancer cells (5/5), while only one lung from mice inoculated with AEPKD MDA-MB-231 cancer cells (1/5) contained a single microscopic metastasis (Fig. 1I, J). Consistently, the difference in total lung metastasis area between mice inoculated with WT and AEPKD cancer cells was strikingly huge (Fig. 1K). IHC staining confirmed the cells formed the metastases originated from MDA-MB-231, as shown by the puncta cytoplasmic staining of human AEP/Legmain (Fig. 1L). In 4T1 cells, cell survival (Fig. S1A) and colony formation were obviously suppressed after AEP knockdown (Fig. S1B, C) in vitro. Lung metastasis in mice was significantly decreased after AEP knockdown (Fig. S1D, E). Together, these results indicate loss of AEP suppresses colony formation under stressed conditions in vitro and metastasis of breast cancer cells in vivo.

### Loss of AEP suppresses autophagosome clearance in breast cancer cells

**Fig. 2:**
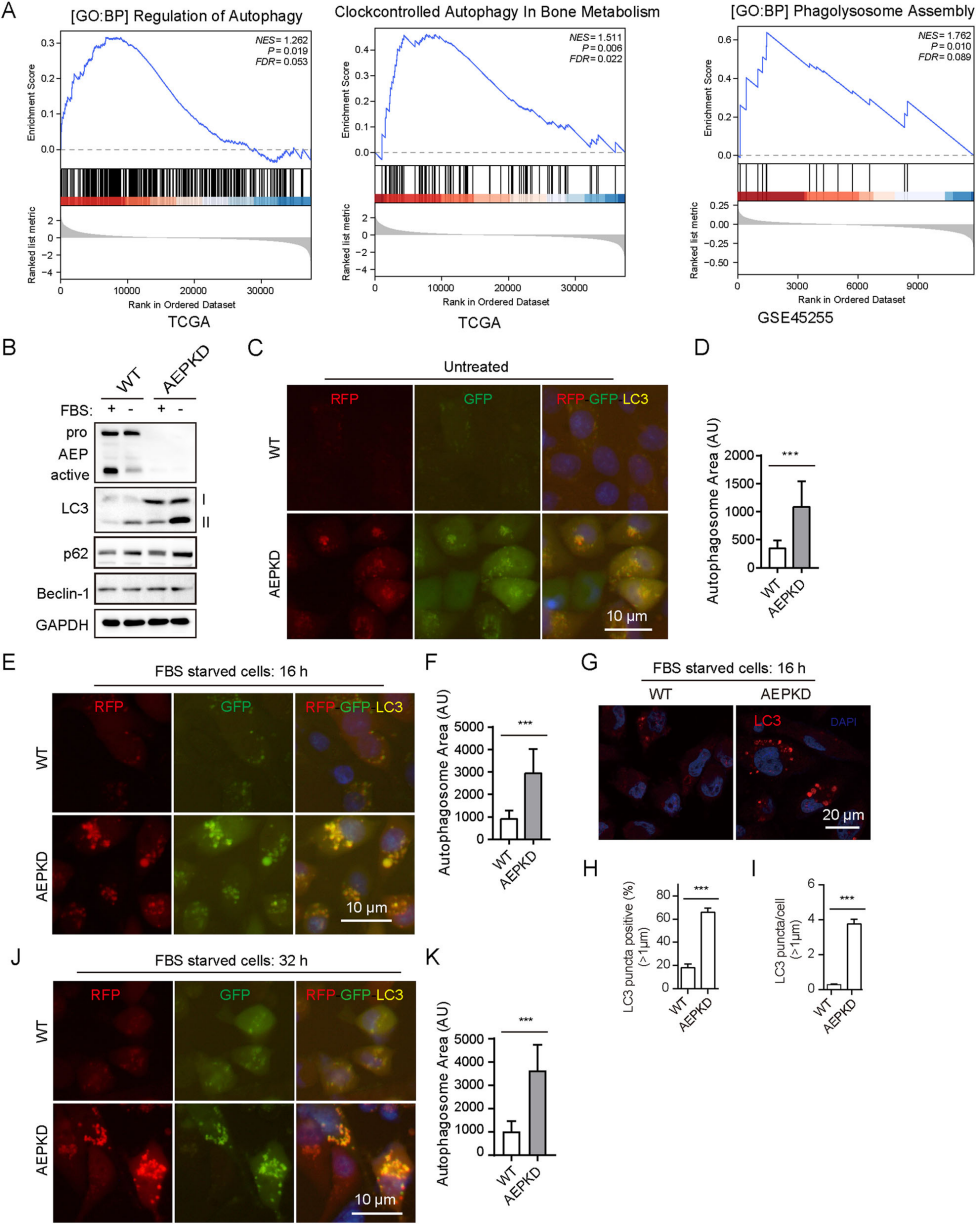
Loss of AEP reduces autophagosome clearance. **A** Gene set enrichment analysis (GSEA) showing enrichment gene sets in AEP-high expressing groups compared with AEP-low groups from TCGA data of BRCA and GSE45255. **B** Immunoblotting analysis of LC3 expression in WT and AEPKD MDA-MB-231 cells. The cells were deprived or not of FBS for 16 h, collected for immunoblotting analysis with antibodies specific to AEP and LC3. GAPDH is loading control. **C** Accumulation of autophagosomes in AEPKD cells. MDA-MB-231 cells were transduced with adenovirus expressing mRFP-GFP tfLC3 for 36 h in full tissue-culture medium and examined live by fluorescence microscopy. Autophagosomes are RFP^+^ and GFP^+^. **D** Quantification of autophagosome area in (**C**). Data are mean ± s.d.; *n* = 50 cells from representative experiments. AU, arbitrary unit. **E** Serum starvation led to accumulation of large autophagosome in AEPKD cells. MDA-MB-231 cells expressing tandem mRFP-GFP-tfLC3 were deprived of FBS for 16 h and examined live by fluorescence microscopy. **F** Quantification of autophagosome area. Data are mean ± s.d.; *n* = 50 cells from representative experiments. **G** Serum-starved cells were fixed, stained with antibody specific to LC3, and examined by confocal microscopy. Quantification of LC3 puncta (≥1 µm) positives cells (%) (**H**) and LC3 puncta (≥1 µm) per cell (**I**). Data are mean ± s.d.; *n* = 50 cells from representative experiments. **J** Persistent autophagosome accumulation in prolong serum-starved AEPKD cells. AEPKD cells were treated as in (**E**) with prolonged serum-starvation of 32 h. **K** Graph showing the autophagosome aera. Results shown are from representative experiments. ***p < 0.001 by two tailed t-test.

To elucidate the biological functions of AEP in BRCA, we compared the gene expression profiles between AEP-low group and AEP-high group by using the RNA sequencing (RNA-seq) data of BRCA patients in the TCGA. Gene set enrichment analysis (GSEA) revealed several gene sets related to autophagy and lysosome function, including Regulation of Autophagy, Autophagy in Bone Metabolism and Phagolysosome Assembly (Fig. 2A). To investigate the potential impact of AEP on autophagy, LC3 expression in tumor cells was observed. We found that loss of AEP increased the expression of LC3 in cells (Fig. 2B). Then breast cancer cells expressing mRFP-GFP tandem fluorescence-tagged LC3 (mRFP-GFP-tfLC3) were used to inspect the effects of AGEs on autophagic flux. Immunofluorescence microscopy examination of live cells expressing mRFP-GFP-tfLC3 showed AEPKD cells contained greater number of autophagosomes (RFP+ and GFP+) than did WT cells (Fig. 2C, D). Inducing autophagosome formation with rapamycin further increased autophagosome accumulation in AEPKD cells (Fig. S2A). We found that serum starvation, which impairs lysosomal degradation, led to accumulation of LC3-II in WT cells, which was accompanied by reduced AEP activation (Fig. 2B). Serum starvation led to even more pronounced accumulation of LC3-II in AEPKD cells (Fig. 2B). Concomitantly, serum-starved AEPKD cells contained remarkably large number and size of autophagosomes, as observed in both live cells expressing mRFP-GFP-tfLC3 (Fig. 2E, F) and fixed cells stained with LC3 antibody (Fig. 2G–I). In addition, prolonged serum starvation led to persistent autophagosome accumulation in AEPKD cells (Fig. 2J, K). Nevertheless, inhibiting lysosome acidification by bafilomycin A1 (BA1) did not further increase autophagosome accumulation in serum starved AEPKD cells (Fig. S2B, C). Together, these results indicate that loss of AEP suppressed autophagy via reducing autophagosome clearance in breast cancer cells.

### Loss of AEP results in aberrant over accumulation of lysosome perinuclear clustering and impairs lysosomal degradation

**Fig. 3:**
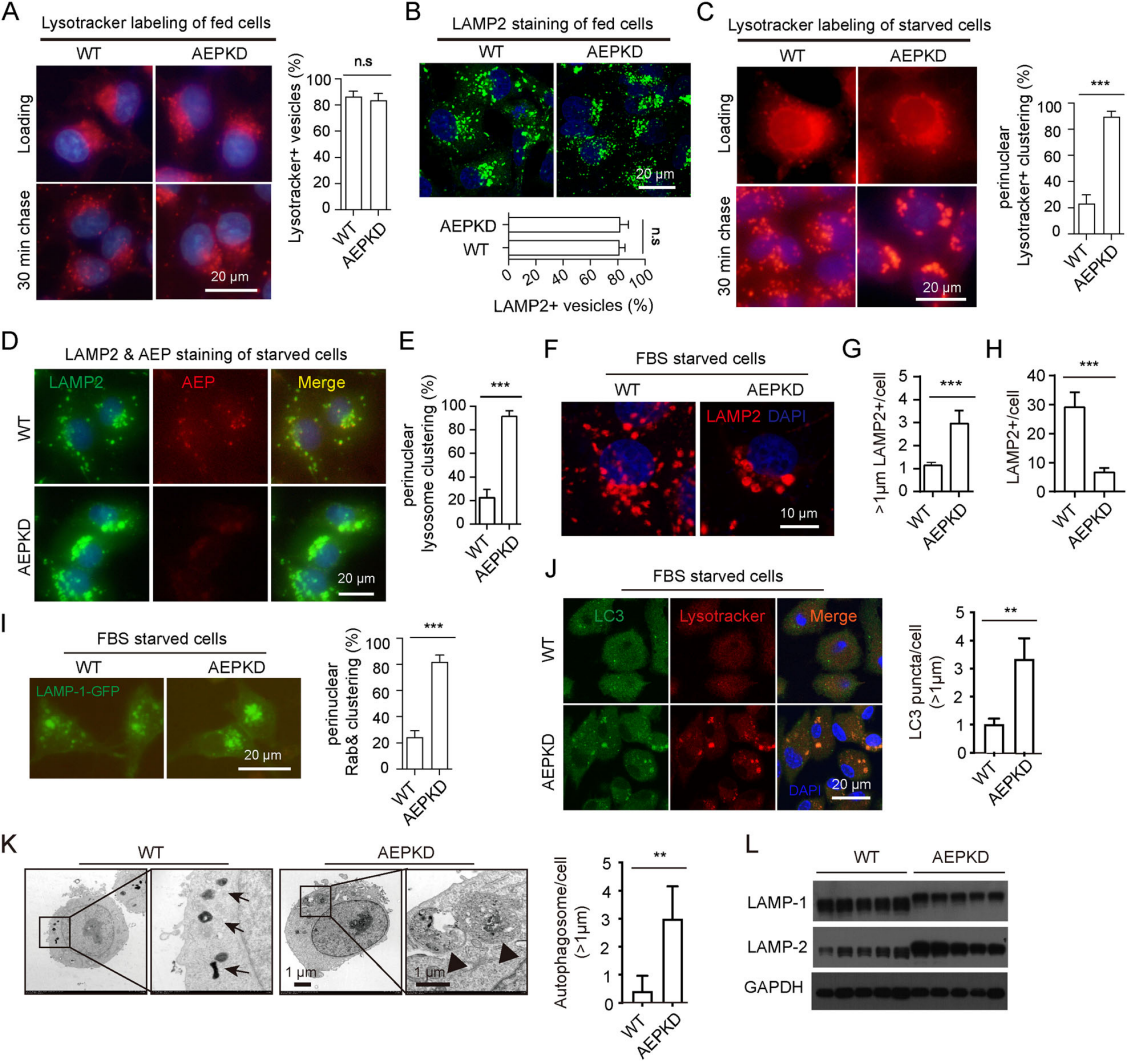
AEP deficiency promotes lysosome perinuclear clustering and fusion and impairs lysosomal degradation. **A** Lysosome positioning in cells in complete culture medium, revealed by Lysotracker labeling and chasing. WT and AEPKD MDA-MB-231 cells were labeled with 2.5 µM Lysotracker for 1 min, washed with cold PBS, and chased for 30 min. **B** Lysosome positioning in cells in complete culture medium, revealed by LAMP2 staining. **C** Elevated lysosome perinuclear clustering and fusion in serum-starved AEPKD cells, revealed by Lysotracker labeling and chasing. **D** Elevated lysosome perinuclear clustering and fusion in serum-starved AEPKD cells, revealed by LAMP2 staining. Cells were double stained with LAMP2 and AEP and examined by fluorescence microscopy. **E** Quantification of cells with predominant perinuclear lysosome clustering in (**D**). Data are mean ± s.d.; *n* = 150 cells from representative experiments. **F** Close examination of lysosome perinuclear localization by confocal microscopy in cells treated as in **D.** Quantification of LAMP2^+^ lysosomes (≥1 µm) (**G**) and total LAMP2^+^ lysosomes (**H**) per cell in (**F**). Data are mean ± s.d.; *n* = 50 cells from representative experiments. **I** Elevated lysosome perinuclear clustering observed in AEPKD cells expressing LAMP-1-GFP. **J** Autophagosomes fused with lysosomes in AEPKD cells. Cells were labeled with Lysotracker, fixed, and stained with LC3 antibody. **K** Analysis of lysosomes in WT and AEPKD cells by transmission electronic microscopy (TEM). Arrows indicate electron-dense lysosomes in WT cells, arrowheads indicate lysosomes/multivesicular bodies with undegraded contents. **L** analysis of LAMP-1 and LAMP-2 in WT and AEPKD cells by immunoblotting. Results shown are from representative experiments. ***p < 0.001 by two-tailed *t*-test.

Autophagosome clearance relies on autophagosome fusion with lysosome to generate an autolysosome for degradation of its content, which was at the last stage of autophagy [36]. This process is largely dependent on lysosome positioning, which results in perinuclear clustering of lysosomes to facilitate autophagosome-lysosome fusion. Thus, we investigated whether the perinuclear clustering of lysosome was affected by AEP knockdown. In full tissue-culture medium, both WT and AEPKD cells showed a similar pattern of lysosome perinuclear localization, with slightly clustered lysosomes in AEPKD cells, as revealed by both Lysotracker labeling/chasing (Fig. 3A) and immunofluorescence staining with lysosome marker LAMP-2 (Fig. 3B). Surprisingly, in serum-starved medium, AEPKD cells showed significantly greater lysosome perinuclear clustering and fusion than did WT cells, as revealed by both Lysotracker labeling/chasing (Fig. 3C) and immunofluorescence staining (Fig. 3D, E). As expected, AEP colocalized with LAMP-2 in WT cells (Fig. 3D). Close examination confirmed that serum-starved AEPKD cells contained larger but fewer lysosomes than did WT cells (Fig. 3F–H). Consistently, examination of live cells expressing GFP-tagged LAMP-1 also demonstrated greater lysosome perinuclear clustering and fusion in AEPKD cells than in WT cells (Fig. 3I). These results indicate that AEP deficiency leads to over accumulation of perinuclear lysosome and less cytoplasmic distribution of lysosome.

Although lysosome perinuclear clustering and fusion is essential for autolysosome formation, lysosome in cytoplasmic was important in maintaining lysosome hemostasis and take role for autolysosome degradation as reported [37]. Therefore, we guest that the impaired lysosome degradation function resulting from aberrant distribution of lysosome might be responsible for reduced autophagosome clearance by AEPKD. As expected, we found that autophagosomes fusion into lysosomes as indicated by colocalization of LC3 with Lysotracker was not affected in AEPKD cells (Fig. 3J). However, TEM examination showed that AEPKD cells contained large autophagosomes fused with lysosomes containing undegraded contents (Fig. 3K). Furthermore, we observed lysosome associated membrane proteins LAMP-1 and LAMP-2 of AEPKD cells showed retarded electrophoretic mobility likely due to over-glycosylation resulting from impaired lysosomal function (Fig. 3L). Similarly, retarded electrophoretic mobility of LAMP2 was observed in AEPKD mouse breast cancer cell line 4T1 (Fig. S3). Together, these results indicate that loss of AEP leads to impaired lysosomal degradation.

### Loss of AEP results in elevated lysosomal pH by modulating V-ATPase stoichiometry/assembly

**Fig. 4:**
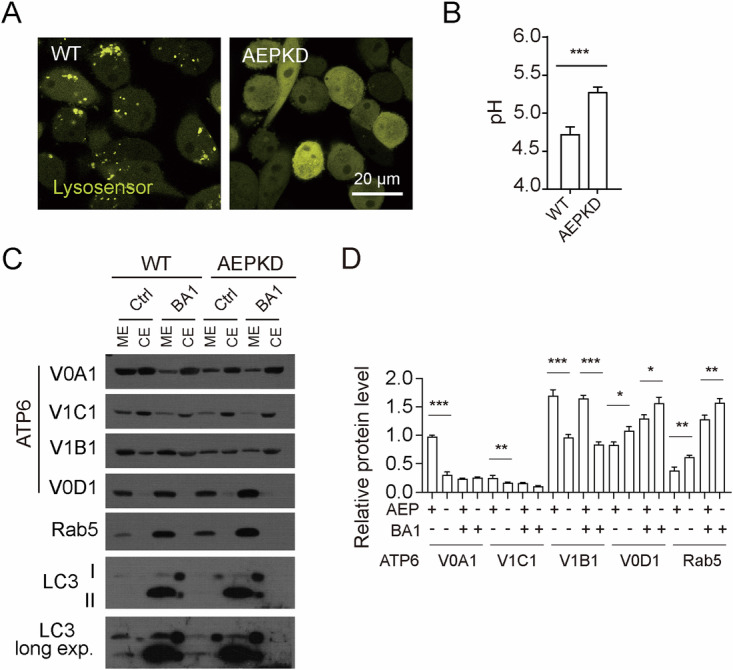
Loss of AEP led to elevated lysosomal pH by modulating V-ATPase stoichiometry/assembly. **A** Lysosensor labeled lysosomes in WT cells but not in AEPKD cells. WT and AEPKD MDA-MB-231 cells were labeled with 2.5 µM lysosensor for 10 min and imaged live. **B** Ratiometric pH measurement showing elevated lysosome pH in AEPKD cells. Data are mean ± s.d.; *n* = 5. **C** Partition of V-ATPase subunits in membrane extract (ME) and cytoplasm extract (CE) in WT and AEPKD cells in the presence or not of 100 nM BA1. ME and CE were analyzed by immunoblotting with indicated antibodies. V-ATPase V0D1, Rab5, and LC3 were used as membrane control. **D** Quantification of protein ME/CE ration in (**C**). Results shown are from representative experiments. **p < 0.01; ***p < 0.001 by two tailed t-test.

We next clarify the mechanism responsible for the aberrant perinuclear distribution of lysosome. It has been reported that elevated intracellular pH upon starvation caused lysosome perinuclear clustering [38]. Confocal microscopy examination of cells labeled with Lysosensor revealed that Lysosensor concentrated in lysosomes in WT cells but undetectable in lysosomes in AEPKD cells (Fig. 4A), suggesting elevated lysosomal pH in the latter. As proof of reliability of the assay, Lysosensor was barely detectable in lysosomes in BA1-treated WT cells (Fig. S4), which is known to elevated lysosomal pH. Ratiometric pH measurement with Lysosensor confirmed higher lysosomal pH in AEPKD cells compared to that in WT cells (5.26 ± 0.12 of AEPKD vs 4.71 ± 0.17 of WT, *p* < 0.001) (Fig. 4B).

The acidic lysosomal lumen is maintained by the lysosomal multi-subunit V-ATPase. To investigate the possible cause of elevated lysosomal pH in AEPKD cells, we performed a fractionation experiment to investigate the assembly of V-ATPase, a regulator of lysosome acidification. We found that more V-ATPase subunits V0A1, V1C1 and V1B1 were partitioned in membrane extracts (ME) in WT cells than in AEPKD cells (Fig. 4C), indicating loss AEP modulated V-ATPase stoichiometry/assembly. As proof of reliability of the assay, treating cells with BA1 reduced partition of V-ATPase subunits V0A1 and V1C1 in MEs, while partition of V-ATPase V1B1 seemed to be unaffected by BA1 treatment (Fig. 4C, D).

### The class IA PI3K regulatory subunit p85 is a substrate of AEP and loss of AEP enhances PI3K activity on endo/lysosomes

**Fig. 5:**
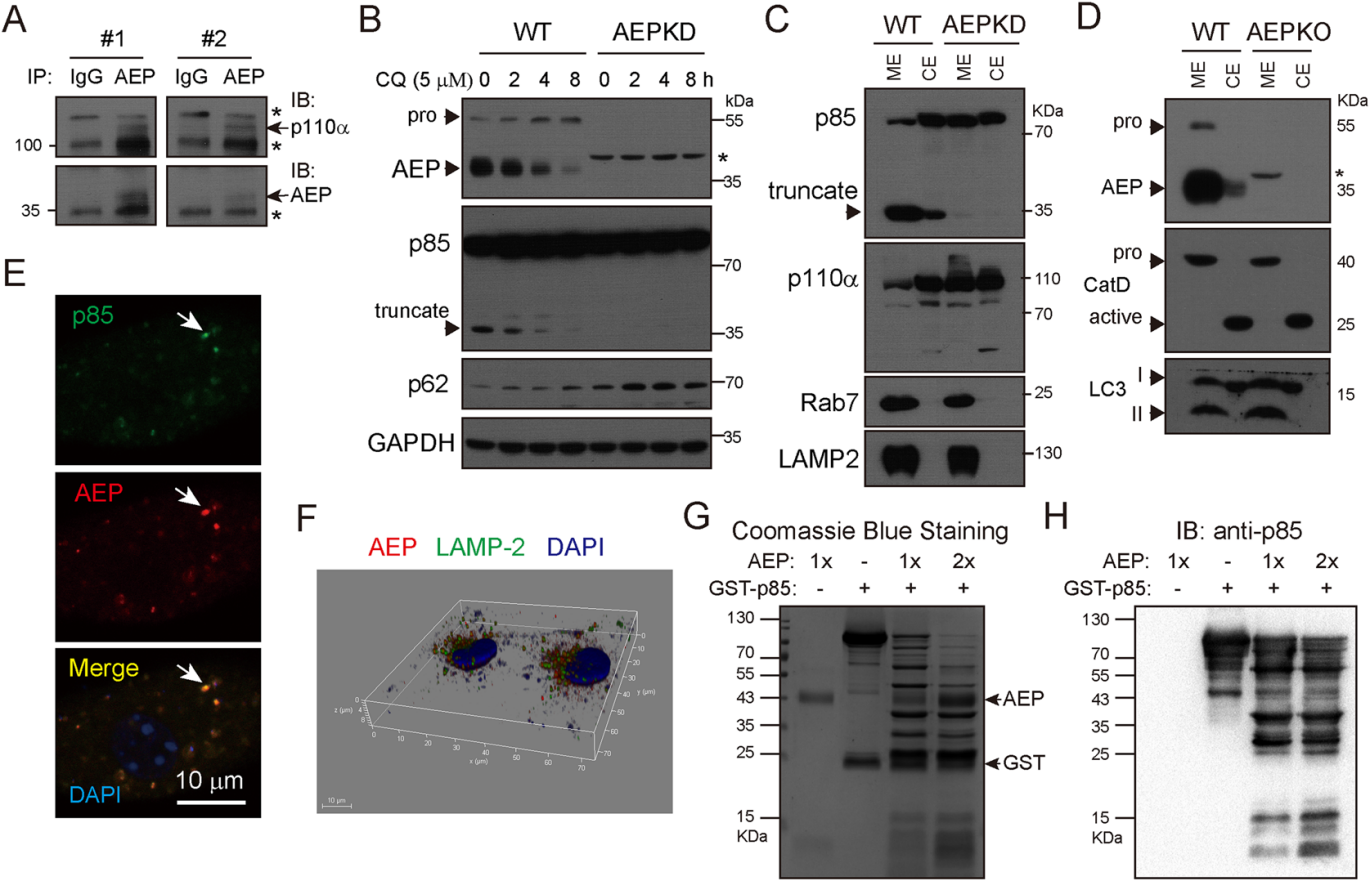
The regulatory subunit p85 of class I_A_ PI3K is a substrate of AEP. **A** p110ɑ coimmunoprecipitated with AEP. Total cell lysates of MDA-MB-231 cells were used for anti-AEP immunoprecipitation and immunoprecipitants were analyzed by immunoblotting with antibodies specific to p110ɑ and AEP. Biological independent replicates. *, non-specific bands. **B** Cleavage of p85 correlated with AEP activation. MDA-MB-231 cells were treated with 5 µM Chloroquine (CQ) for indicated times. AEP activation and p85 cleavage were analyzed by immunoblotting with antibodies specific to AEP, p85, and p62. **C**, **D** Subcellular fractionation showing membrane localization of p85 truncate and AEP. ME and CE were analyzed by immunoblotting with indicated antibodies. Rab7, LAMP2, LC3, and cathepsin D are loading controls. The immunoblots shown are from representative experiments. **E** MDA-MB-231 cells were immunostained with antibodies specific to p85 and AEP, and examined by confocal microscopy. Arrow denotes p85 and AEP colocalization. **F** AEP localized on the outer membrane of and inside lysosomes. 3D images of cells stained with LAMP02 and AEP were re-constructed from confocal microscopy serial optical Z-sections. AEP cleaved p85 in vitro. GST-p85 was incubated with increasing amount of AEP in vitro cleavage experiment. Cleavage of p85 was visualized by Coomassie blue staining (**G**) or by immunoblotting with antibody specific to p85 (**H**) after SDS-PAGE separation. The results shown are from representative experiments.

To investigate the molecular bases by which AEP regulates lysosomal function, we performed co-immunoprecipitation with antibody specific to AEP/Legumen. Interestingly, by IP-MS, we found P13K was interacted with AEP. Recently, it has been suggested that PI3K can be activated on endo/lysosomes [37], however, whether it play a role in mediating lysosome function has not been clarified yet. We detect p110ɑ but not p85 class IA of PI3K in AEP/Legumain immunoprecipitants by immunoblotting (Fig. 5A), probably due to degradation of p85 by AEP. Indeed, p85 truncation was detected in the lysates of WT cells by immunoblotting (Fig. 5B). The levels of p85 truncation were proportional to the levels of AEP activation in response to Chloroquine treatment (Fig. 5B). Subcellular fractionation experiment showed that most of the p85 truncate was found in the ME of WT cells (Fig. 5C). Meanwhile, the majority of AEP was also found in the ME (Fig. 5D). The interaction between AEP and p110α was also conformed in 4T1 cells (Fig. S5A). We also showed that cleavage of p85 was tightly correlated with activation of AEP, which was regulated by CQ (Fig. S5B). Truncated p85 was found on the membrane fractionation (Fig. S5C), where AEP was also found in 4T1 cells (Fig. S5D). Confocal microscopy examination revealed that p85 colocalized with AEP on vesicles (Fig. 5E). In addition, 3D reconstruction of confocal images showed substantial AEP could be found on the outer membrane of lysosomes, which might help to explain how AEP interacted with p85 (Fig. 5F and Video Still). Moreover, in vitro enzyme cleavage experiment showed that p85 was cleaved by AEP in a dose-dependent manner (Fig. 5G, H). P85 degradation by AEP was accompanied by reduced levels of p110ɑ (Fig. 5C). Together, these results identify p85 of class IA PI3K as a substrate of AEP.

**Fig. 6:**
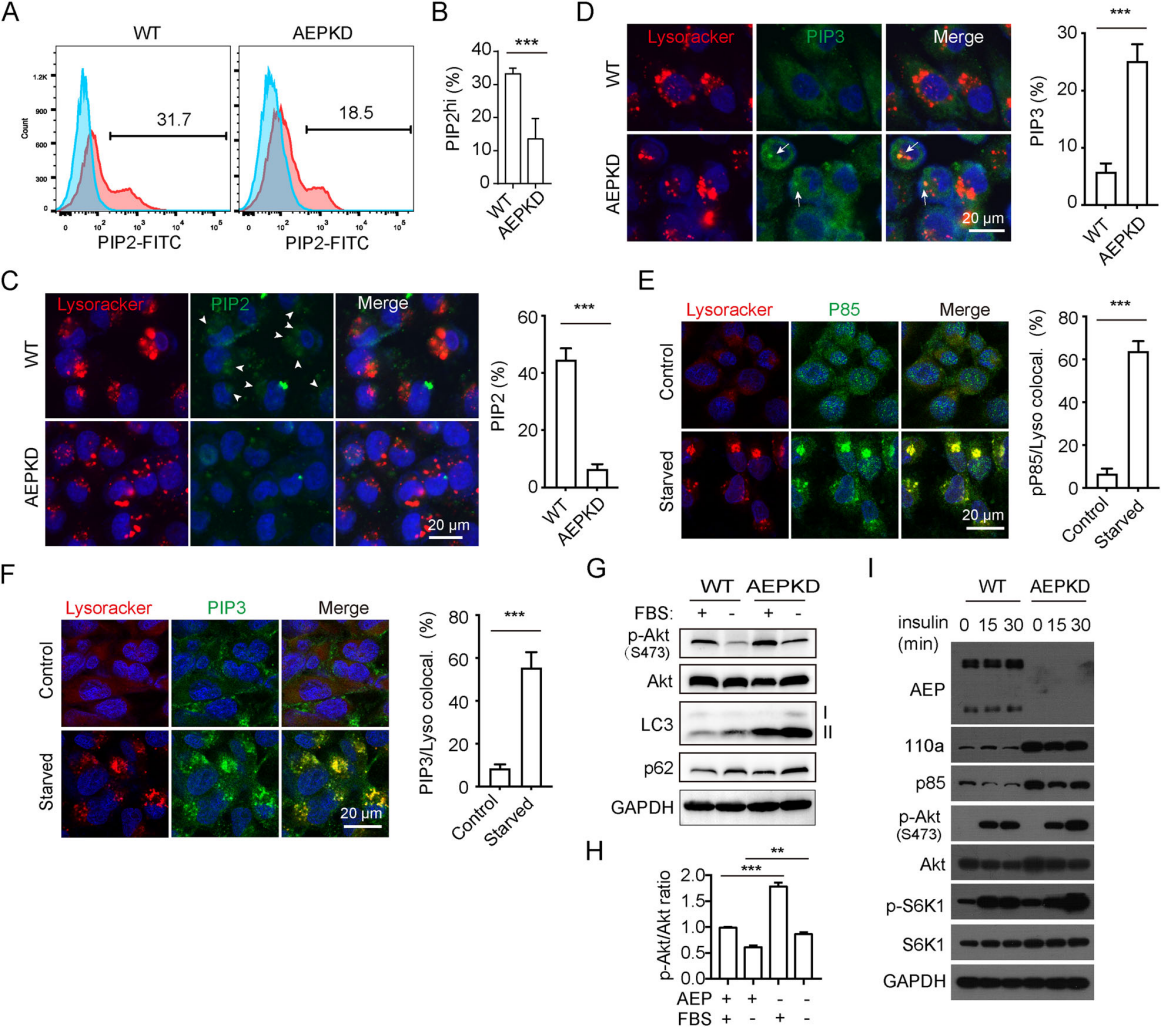
Loss of AEP enhances PI3K activity on endo/lysosomes. **A** Flow cytometry analysis of PIP_2_ levels in WT and AEPKD MDA-MB-231 cells immunostained with antibody specific to PIP_2_. **B** Quantification of percentage of PIP2^hi^ cells treated as in (**A**). Data are mean ± s.d.; *n* = 3. Confocal microscopy analysis showing higher PIP_2_ levels on endo/lysosomes in WT cells and higher PIP_3_ levels on endo/lysosomes in AEPKD cells. MDA-MB-231 cells were labeled with 2.5 µM Lysotracker for 1 h, and stained with antibodies specific to PIP_2_ (**C**) or PIP_3_ (**D**). Arrow heads and arrows indicate colocalization of Lysotracker with PIP_2_ or PIP_3_ on vesicles respectively. Serum starvation significantly increased PI3K and PIP_3_ on endo/lysosomes in AEPKD cells. Cells were deprived of FBS for 16 h, labeled with 2.5 µM Lysotracker for 1 min, chased for 30 min, immunostained with antibodies specific to p85 (**E**) or PIP_3_ (**F**). **G** Immunoblotting analysis showing higher PI3K activity in AEPKD cells. MDA-MB-231 cells were deprived of FBS or not for 16 h. Cell lysates were analyzed by immunoblotting with antibodies specific to phosphorylated Akt, Akt and indicated. **H** Densitometry analysis of p-Akt and Akt in cells treated as in (**G**). Data are mean ± s.d.; *n* = 3 independent experiments. **I** Enhanced recovery of PI3K activity in AEPKD cells after insulin stimulation. MDA-MB-231 cells were deprived of FBS for 4 h, stimulated with 100 ng/mL insulin for indicated times. Cell lysates were analyzed by immunoblotting with antibodies as indicated. Results shown are from representative experiments. **p < 0.01; ***p < 0.001 by two-tailed t-test.

We next investigated the effect of AEP on PI3K activity on endo/lysosomes. Activated class I PI3Ks convert PtdIns (4,5) P2 (PIP2) to PtdIns (3,4,5) P3 (PIP3) on cellular membranes [39–41]. To compare PI3K activities, we analyzed PIP2 and PIP3 levels in WT and AEPKD MDA-MB-231 cells. Flow cytometry analysis showed that the percentage of PIP2hi cells in WT cells was significantly higher than that in AEPKD cells (Fig. 6A, B). Because the low levels of PIP3 in cells, we could not effectively detect PIP3 by flow cytometry. Confocal microscopy study identified PIP2 on Lysotracker labeled vesicles in WT cells but not in AEPKD cells (Fig. 6C). On the other hand, we observed low levels of PIP3 on Lysotracker labeled vesicles in AEPKD cells but not in WT cells (Fig. 6D). Serum starvation led to remarkable concentration of PI3K on Lysotracker labeled vesicles in AEPKD cells (Fig. 6E). Concomitantly, higher PIP3 levels were found on Lysotracker labeled vesicles in AEPKD cells (Fig. 6F). In agreement, PI3K activity was higher in AEPKD cells than in WT cells, as demonstrated by phosphorylation of Akt at Thr473 (Fig. 6G). Moreover, PI3K activity was more resistant to serum starvation in AEPKD cells than in WT cells (Fig. 6G, H). On the other hand, insulin stimulation of serum-starved cells for up to 30 min led to stronger recovery of PI3K activity in AEPKD cells than in WT cells (Fig. 6I). Noteworthy, AEPKD cells also contained higher levels of p85 and p110ɑ than WT cells (Fig. 6I). Together, these results indicate that loss of AEP enhances PI3K activity on endo/lysosomes.

We then used siRNA to knockdown expression of subunits of PI3K, including PIK3CA, PIK3CB, PIK3R1, and PIK3R2. We successfully knocked-down expression of PIK3CA, PIK3CB, PIK3R1 in both WT and AEPKD MDA-MB-231 cells. Acute knockdown of PI3K expression caused impaired lysosome degradation in AEP WT cells (Fig. S6A, B), while had little effect on lysosome degradation in AEPKD cells (Fig. S6C, Fig. 6D, E), indicating PI3K had feedback effect on AEP and lysosome degradation.

### Inhibition of endo/lysosomal PI3K activity leads to lysosome acidification and enhances lysosomal degradation

**Fig. 7:**
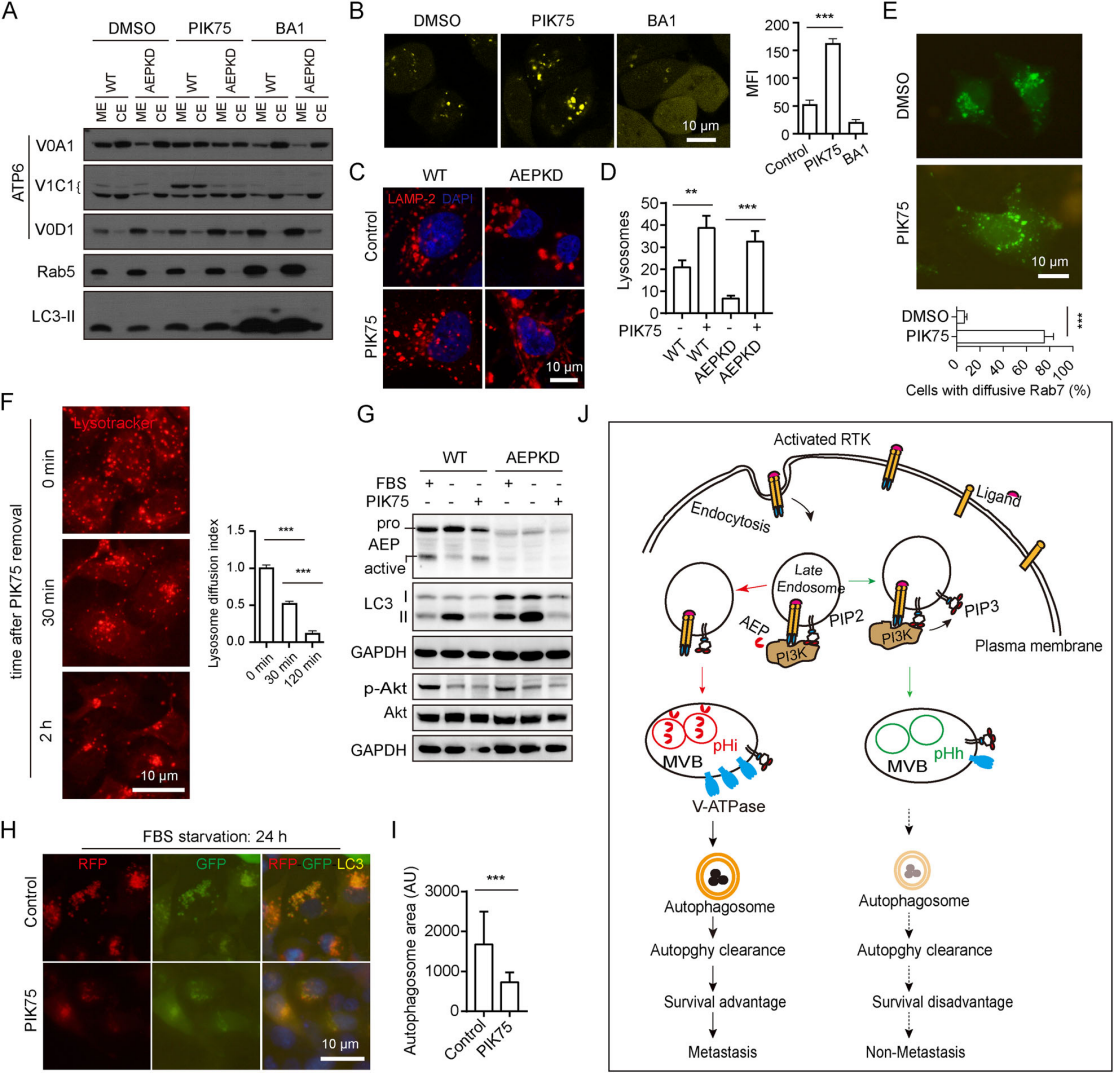
Inhibition of endo/lysosomal PI3K activity leads to lysosome acidification and enhances lysosomal degradation. **A** Partition of V-ATPase proteins in MEs and CEs after inhibition of PI3K. Cells were treated without or with 100 nM PIK75 or BA1 overnight. **B** Confocal microscopy analysis of cells labeled with Lysosensor. Cells were labeled with 2.5 µM Lysosensor for 10 min before imaged live. **C** PIK75 treatment prevented lysosome perinuclear clustering and fusion in serum-starved cells. Cells were deprived of FBS for 24 h, in the presence or absence of 100 nM PIK75 for the last 5 h, and stained with LAMP-2 antibody. **D** Quantification of LAMP-2^+^ lysosomes/cell in (**C**). Data are mean ± s.d.; *n* = 50 cells from representative experiments. **E** Live-cell imaging of AEPKD cells expressing LAMP-1-GFP showing PIK75 treatment prevented lysosome clustering. **F** Removal of PIK75 resulted in lysosome perinuclear re-aggregation in a time-dependent manner. AEPKD MDA-MB-231 cells were treated as in (**C**). PIK75 was removed by washing with cold PBS. Cells were then labeled with 2.5 µM Lysotracker for 1 min, chased for indicated times. **G** PIK75 treatment enhanced clearance of LC3-II. WT and AEPKD MDA-MB-231 cells were treated as in (**C**). Cell lysates were analyzed by immunoblotting with antibodies as indicated. **H** PIK75 treatment promoted autophagosome clearance. AEPKD MDA-MB-231 cells expressing mRFP-GFP-tfLC3 were treated as in (**C**), and imaged live by fluorescence microscopy. **I** Quantification of autophagosome area in (**H**). AU arbitrary unit. Data are mean ± s.d.; n = 50 cells from representative experiments. **J** Model depicting the effect of AEP in modulating lysosomal function and autophagy in BRCA. Results shown are from representative experiments. **p < 0.01; ***p < 0.001 by two-tailed t-test.

We next aim to verify whether PI3Ks was responsible for the function of AEP knockdown in lysosome degradation. Based on the above results, we reasoned that inhibiting endo/lysosomal PI3K activity would lead to lysosome acidification. We screened several PI3K inhibitors and identified PIK75 as a potent PI3K inhibitor (Fig. S7A), which even led to degradation of p110ɑ and p85 in WT cells at modest dosage (Fig. S7A). PIK75 treatment led to increased expression and partition of V-ATPase V0A1 and V1C1 to endo-membranes in both WT and AEPKD cells (Fig. 7A). As expected, PIK75 treatment led to lysosome acidification, as shown by remarkable concentration of Lysosensor in lysosomes of PIK75 treated cells (Fig. 7B). In contrast, Lysosensor was just detectable in lysosomes in untreated control cells and barely detectable in lysosomes in BA1 treated cells (Fig. 7B).

Consistent with intracellular pH regulating lysosome positioning [38], we found that PIK75 treatment prevented lysosome perinuclear clustering/fusion induced by serum starvation in both WT and AEPKD cells (Fig. 7C, D). PIK75 treatment not only resulted in diffusive peripheral distribution of lysosomes but also increased the number of lysosomes in cells (Fig. 7C, D). Similar results were observed in live cells expressing LAMP-1-GFP (Fig. 7E). On the other hand, removal of PIK75 from culture medium resulted in lysosome re-clustering in a time-dependent manner (Fig. 7F). Meanwhile, PIK75 treatment resulted in clearance of LC3-II (Fig. 7G) and p62 (Fig. S7A–C), and autophagosomes in serum-starved cells (Fig. 7H, I). Notably, PIK75 treatment restored AEP activation (Fig. 7G). In contrast, removal of PIK75 from culture medium resulted in autophagosome re-accumulation in cells (Fig. S7D, E). As comparison, Rapamycin did not induce autophagosome clearance (Fig. 2D), or the clearance of p62 (Fig. S7C). Together, these results indicate that inhibition of endo/lysosomal PI3K leads to lysosome acidification and enhances lysosomal degradation.

## Discussion

It has been shown previously that lysosomes of renal proximal tubular cells in AEP-deficient mice are enlarged and merged with late endosomes [30]. In this study, we extend this finding in breast cancer cells by demonstrating that loss of AEP promotes lysosome perinuclear aggregation and fusion due to defective lysosome acidification. We propose that elevated endo/lysosomal class IA PI3K activity in AEPKD cells altered membrane phosphoinositide composition which in turn modulated lysosomal V-ATPase assembly. We propose that overexpression of AEP in such as cancer cells could enhance lysosome degradation via lysosome acidification, which in turn enhances catabolic response in cells.

Triple-negative breast cancer (TNBC) represents an aggressive, metastatic, and notably challenging subtype of breast cancers, posing significant therapeutic hurdles [42]. Currently, chemotherapy stands as the sole treatment modality for TNBC, albeit with limited efficacy, resulting from the inherent heterogeneity of TNBC, which encompasses diverse oncogenic drivers, or the development of resistance to existing chemotherapeutics [43–45]. Consequently, there is a pressing need to explore novel therapeutic avenues, targeting alternative molecular mechanisms, for the effective treatment of TNBC. Blockade of Asparagine Endopeptidase with specific inhibitors has been found suppress cancer metastasis [46]. Consistent with the previous report that combination use of small molecule inhibitors of Asparagine endopeptidase and epirubicin could prevent bone metastasis in breast cancer [47]. Our study suggests that AEP might function as a potential target to be used in the metastasis of breast cancer.

Normally, agonist-stimulated PI3K activation confines p85 and p110ɑ to the plasma membrane [48]. Interestingly, recent studies have suggested that PI3K can be activated on endo/lysosomes [38], however, how its activity on endo/lysosomes is regulated and whether it plays a role in regulating endo/lysosome homeostasis is unclear. Here, we demonstrate for the first time the role of PI3Ks in regulating V-ATPase assembly on endo/lysosomes, and further elucidate their regulation by AEP in breast cancers. Enhanced endocytosis may result in the translocation of residual activated RTKs into late endosomes, possibly facilitated by other factors such as MAP4 [37], PI3Ks are recruited to RTKs on endosomal membranes. In WT cells, endosomal PI3Ks are tightly controlled by AEP when lysosomes “kiss” late endosomes. AEP may degrade p85 on the cytoplasmic side of endosomal membranes. In AEP deficient cells, however, elevated endosomal PI3Ks upon nutrient starvation, i.e. “endocytosis-stimulated PI3K activation”, can proceed to convert PIP2 to PIP3 on late endosomes/lysosomes. How membrane PIP3 levels regulates V-ATPase assembly warrants further investigation. Nevertheless, our results are consistent with a previous study which has shown that nutrient starvation leads to elevated intracellular pH and perinuclear clustering of lysosomes [38].

In line with our findings that V-ATPase can regulate lysosomal acidification in breast cancer cells, a similar correlation between V-ATPase activation and lysosomal acidification has been observed in other cell types. During their maturation process, dendritic cells acquire an enhanced ability to form and accumulate peptide-MHC (major histocompatibility complex) class II, a process that necessitates the activation of V-ATPase and lysosomal acidification [49]. Interestingly, AEP may fulfill these two roles at the same time. On one hand, AEP can process antigen for MCH II presentation [16]. On the other hand, AEP can regulate lysosomal acidification as shown by our data here.

PI3K activating PI3KCA mutations are reported to be present in about 25% of breast cancer [50, 51]. In such circumstances, AEP may help to sever excessive endo/lysosomal PI3K activity and thus render cancer cells survival advantage by buoying cellular metabolism as mentioned above. Importantly, we have screened several PI3K inhibitors and identified PIK75 as a potent PI3K inhibitor taken effect on endosomal PI3K, which even led to degradation of p110ɑ and p85 in tumor cells at modest dosage. Its effect on endosomal PI3K might indicate its specific effect on regulating autophagy especially in tumor cells with high AEP expression.

In summary, we have discovered a novel mechanism by which the AEP/PI3K axis regulates lysosome homeostasis and function, potentially representing a promising target for inhibiting metabolic adaptations in cancer.

## Supplementary information

Supplemental figures

## Data Availability

The data are available within the Article, Supplementary Information, or available from the authors upon request. Source data are provided with this paper.

## References

[1] Harbeck N, Gnant M. Breast cancer. Lancet. 2017;389:1134–50. 27865536 10.1016/S0140-6736(16)31891-8

[2] Katsura C, Ogunmwonyi I, Kankam HK, Saha S. Breast cancer: presentation, investigation and management. Br J Hosp Med. 2022;83:1–7. 10.12968/hmed.2021.0459 35243878

[3] Li X, He S, Ma B. Autophagy and autophagy-related proteins in cancer. Mol Cancer. 2020;19:12. 31969156 10.1186/s12943-020-1138-4 PMC6975070

[4] Hou J, Han Z, Zhao N, Wei L. Autophagy and tumour metastasis. Adv Exp Med Biol. 2020;1207:315–38. 32671757 10.1007/978-981-15-4272-5_22

[5] Levy JMM, Towers CG, Thorburn A. Targeting autophagy in cancer. Nat Rev Cancer. 2017;17:528–42. 28751651 10.1038/nrc.2017.53 PMC5975367

[6] Chen JM, Dando PM, Rawlings ND, Brown MA, Young NE, Stevens RA, et al. Cloning, isolation, and characterization of mammalian legumain, an asparaginyl endopeptidase. J Biol Chem. 1997;272:8090–8. 9065484 10.1074/jbc.272.12.8090

[7] Zhang X, Tang L, Zhang Z. ADAM10 and ADAM17 are degraded by lysosomal pathway via asparagine endopeptidase. Biochem Biophys Res Commun. 2021;537:15–21. 33383559 10.1016/j.bbrc.2020.12.063

[8] Dall E, Brandstetter H. Mechanistic and structural studies on legumain explain its zymogenicity, distinct activation pathways, and regulation. Proc Natl Acad Sci USA. 2013;110:10940–5. 23776206 10.1073/pnas.1300686110 PMC3703970

[9] Li DN, Matthews SP, Antoniou AN, Mazzeo D, Watts C. Multistep autoactivation of asparaginyl endopeptidase in vitro and in vivo. J Biol Chem. 2003;278:38980–90. 12860980 10.1074/jbc.M305930200

[10] Halfon S, Patel S, Vega F, Zurawski S, Zurawski G. Autocatalytic activation of human legumain at aspartic acid residues. FEBS Lett. 1998;438:114–8. 9821970 10.1016/s0014-5793(98)01281-2

[11] Zhao L, Hua T, Crowley C, Ru H, Ni X, Shaw N, et al. Structural analysis of asparaginyl endopeptidase reveals the activation mechanism and a reversible intermediate maturation stage. Cell Res. 2014;24:344–58. 24407422 10.1038/cr.2014.4 PMC3945893

[12] Chen JM, Dando PM, Stevens RA, Fortunato M, Barrett AJ. Cloning and expression of mouse legumain, a lysosomal endopeptidase. Biochem J. 1998;335(Pt 1):111–7. 9742219 10.1042/bj3350111 PMC1219758

[13] Miller G, Matthews SP, Reinheckel T, Fleming S, Watts C. Asparagine endopeptidase is required for normal kidney physiology and homeostasis. FASEB J. 2011;25:1606–17. 21292981 10.1096/fj.10-172312

[14] Clerin V, Shih HH, Deng N, Hebert G, Resmini C, Shields KM, et al. Expression of the cysteine protease legumain in vascular lesions and functional implications in atherogenesis. Atherosclerosis. 2008;201:53–66. 18377911 10.1016/j.atherosclerosis.2008.01.016

[15] Choi SJ, Reddy SV, Devlin RD, Menaa C, Chung H, Boyce BF, et al. Identification of human asparaginyl endopeptidase (legumain) as an inhibitor of osteoclast formation and bone resorption. J Biol Chem. 1999;274:27747–53. 10488118 10.1074/jbc.274.39.27747

[16] Manoury B, Hewitt EW, Morrice N, Dando PM, Barrett AJ, Watts C. An asparaginyl endopeptidase processes a microbial antigen for class II MHC presentation. Nature. 1998;396:695–9. 9872320 10.1038/25379

[17] Sepulveda FE, Maschalidi S, Colisson R, Heslop L, Ghirelli C, Sakka E, et al. Critical role for asparagine endopeptidase in endocytic toll-like receptor signaling in dendritic cells. Immunity. 2009;31:737–48. 19879164 10.1016/j.immuni.2009.09.013

[18] Zhang Z, Song M, Liu X, Kang SS, Kwon IS, Duong DM, et al. Cleavage of tau by asparagine endopeptidase mediates the neurofibrillary pathology in Alzheimer’s disease. Nat Med. 2014;20:1254–62. 25326800 10.1038/nm.3700 PMC4224595

[19] Zhang Z, Kang SS, Liu X, Ahn EH, Zhang Z, He L, et al. Asparagine endopeptidase cleaves alpha-synuclein and mediates pathologic activities in Parkinson’s disease. Nat Struct Mol Biol. 2017;24:632–42. 28671665 10.1038/nsmb.3433 PMC5871868

[20] Basurto-Islas G, Grundke-Iqbal I, Tung YC, Liu F, Iqbal K. Activation of asparaginyl endopeptidase leads to Tau hyperphosphorylation in Alzheimer disease. J Biol Chem. 2013;288:17495–507. 23640887 10.1074/jbc.M112.446070 PMC3682549

[21] Patel N, Krishnan S, Offman MN, Krol M, Moss CX, Leighton C, et al. A dyad of lymphoblastic lysosomal cysteine proteases degrades the antileukemic drug L-asparaginase. J Clin Investig. 2009;119:1964–73. 19509471 10.1172/JCI37977 PMC2701869

[22] Murthy RV, Arbman G, Gao J, Roodman GD, Sun XF. Legumain expression in relation to clinicopathologic and biological variables in colorectal cancer. Clin Cancer Res. 2005;11:2293–9. 15788679 10.1158/1078-0432.CCR-04-1642

[23] Haugen MH, Boye K, Nesland JM, Pettersen SJ, Egeland EV, Tamhane T, et al. High expression of the cysteine proteinase legumain in colorectal cancer-implications for therapeutic targeting. Eur J Cancer. 2015;51:9–17. 25466510 10.1016/j.ejca.2014.10.020

[24] Haugen MH, Johansen HT, Pettersen SJ, Solberg R, Brix K, Flatmark K, et al. Nuclear legumain activity in colorectal cancer. PloS One. 2013;8:e52980. 23326369 10.1371/journal.pone.0052980 PMC3542341

[25] Wang L, Chen S, Zhang M, Li N, Chen Y, Su W, et al. Legumain: a biomarker for diagnosis and prognosis of human ovarian cancer. J Cell Biochem. 2012;113:2679–86. 22441772 10.1002/jcb.24143

[26] Wang Y, Zhang S, Wang H, Cui Y, Wang Z, Cheng X, et al. High level of legumain was correlated with worse prognosis and peritoneal metastasis in gastric cancer patients. Front Oncol. 2020;10:966. 32766126 10.3389/fonc.2020.00966 PMC7378441

[27] Wang H, Chen B, Lin Y, Zhou Y, Li X. Legumain promotes gastric cancer progression through tumor-associated macrophages in vitro and in vivo. Int J Biol Sci. 2020;16:172–80. 31892854 10.7150/ijbs.36467 PMC6930372

[28] Gawenda J, Traub F, Luck HJ, Kreipe H, von Wasielewski R. Legumain expression as a prognostic factor in breast cancer patients. Breast Cancer Res Treat. 2007;102:1–6. 17028987 10.1007/s10549-006-9311-z

[29] Lin Y, Qiu Y, Xu C, Liu Q, Peng B, Kaufmann GF, et al. Functional role of asparaginyl endopeptidase ubiquitination by TRAF6 in tumor invasion and metastasis. J Natl Cancer Inst. 2014;106:dju012. 24610907 10.1093/jnci/dju012

[30] Shirahama-Noda K, Yamamoto A, Sugihara K, Hashimoto N, Asano M, Nishimura M, et al. Biosynthetic processing of cathepsins and lysosomal degradation are abolished in asparaginyl endopeptidase-deficient mice. J Biol Chem. 2003;278:33194–9. 12775715 10.1074/jbc.M302742200

[31] Diwu Z, Chen CS, Zhang C, Klaubert DH, Haugland RP. A novel acidotropic pH indicator and its potential application in labeling acidic organelles of live cells. Chem Biol. 1999;6:411–8. 10381401 10.1016/s1074-5521(99)80059-3

[32] Uhlen M, Fagerberg L, Hallstrom BM, Lindskog C, Oksvold P, Mardinoglu A, et al. Proteomics. Tissue-based map of the human proteome. Science. 2015;347:1260419. 25613900 10.1126/science.1260419

[33] Uhlen M, Zhang C, Lee S, Sjostedt E, Fagerberg L, Bidkhori G, et al. A pathology atlas of the human cancer transcriptome. Science. 2017;357:eaan2507. 10.1126/science.aan2507 28818916

[34] Uhlen M, Bjorling E, Agaton C, Szigyarto CA, Amini B, Andersen E, et al. A human protein atlas for normal and cancer tissues based on antibody proteomics. Mol Cell Proteom. 2005;4:1920–32. 10.1074/mcp.M500279-MCP200 16127175

[35] Chan CB, Abe M, Hashimoto N, Hao C, Williams IR, Liu X, et al. Mice lacking asparaginyl endopeptidase develop disorders resembling hemophagocytic syndrome. Proc Natl Acad Sci USA. 2009;106:468–73. 19106291 10.1073/pnas.0809824105 PMC2626726

[36] Pohl C, Dikic I. Cellular quality control by the ubiquitin-proteasome system and autophagy. Science. 2019;366:818–22. 31727826 10.1126/science.aax3769

[37] Thapa N, Chen M, Horn HT, Choi S, Wen T, Anderson RA. Phosphatidylinositol-3-OH kinase signalling is spatially organized at endosomal compartments by microtubule-associated protein 4. Nat Cell Biol. 2020;22:1357–70. 33139939 10.1038/s41556-020-00596-4 PMC8647654

[38] Korolchuk VI, Saiki S, Lichtenberg M, Siddiqi FH, Roberts EA, Imarisio S, et al. Lysosomal positioning coordinates cellular nutrient responses. Nat Cell Biol. 2011;13:453–60. 21394080 10.1038/ncb2204 PMC3071334

[39] Vivanco I, Sawyers CL. The phosphatidylinositol 3-Kinase AKT pathway in human cancer. Nat Rev Cancer. 2002;2:489–501. 12094235 10.1038/nrc839

[40] Vanhaesebroeck B, Stephens L, Hawkins P. PI3K signalling: the path to discovery and understanding. Nat Rev Mol Cell Biol. 2012;13:195–203. 22358332 10.1038/nrm3290

[41] Bilanges B, Posor Y, Vanhaesebroeck B. PI3K isoforms in cell signalling and vesicle trafficking. Nat Rev Mol Cell Biol. 2019;20:515–34. 31110302 10.1038/s41580-019-0129-z

[42] Yao H, He G, Yan S, Chen C, Song L, Rosol TJ, et al. Triple-negative breast cancer: is there a treatment on the horizon? Oncotarget. 2017;8:1913–24. 27765921 10.18632/oncotarget.12284 PMC5352107

[43] Wein L, Loi S. Mechanisms of resistance of chemotherapy in early-stage triple-negative breast cancer (TNBC). Breast. 2017;34(Suppl 1):S27–30. 28668293 10.1016/j.breast.2017.06.023

[44] Ferrari P, Scatena C, Ghilli M, Bargagna I, Lorenzini G, Nicolini A. Molecular mechanisms, biomarkers and emerging therapies for chemotherapy resistant TNBC. Int J Mol Sci. 2022;23:1665. 10.3390/ijms23031665 PMC8836182 35163586

[45] Biswas A, Dalton MJ. Postperinatal mortality in a health district with a garrison town. BMJ. 1988;297:1195. 3144351 10.1136/bmj.297.6657.1195 PMC1834978

[46] Qi Q, Obianyo O, Du Y, Fu H, Li S, Ye K. Blockade of asparagine endopeptidase inhibits cancer metastasis. J Med Chem. 2017;60:7244–55. 28820254 10.1021/acs.jmedchem.7b00228 PMC5871875

[47] Chen J, Xu W, Song K, Da LT, Zhang X, Lin M, et al. Legumain inhibitor prevents breast cancer bone metastasis by attenuating osteoclast differentiation and function. Bone. 2023;169:116680. 36702335 10.1016/j.bone.2023.116680

[48] Lee JY, Engelman JA, Cantley LC. Biochemistry. PI3K charges ahead. Science. 2007;317:206–7. 17626872 10.1126/science.1146073

[49] Trombetta ES, Ebersold M, Garrett W, Pypaert M, Mellman I. Activation of lysosomal function during dendritic cell maturation. Science. 2003;299:1400–3. 12610307 10.1126/science.1080106

[50] Loi S, Haibe-Kains B, Majjaj S, Lallemand F, Durbecq V, Larsimont D, et al. PIK3CA mutations associated with gene signature of low mTORC1 signaling and better outcomes in estrogen receptor-positive breast cancer. Proc Natl Acad Sci USA. 2010;107:10208–13. 20479250 10.1073/pnas.0907011107 PMC2890442

[51] Ellis H, Ma CX. PI3K inhibitors in breast cancer therapy. Curr Oncol Rep. 2019;21:110. 31828441 10.1007/s11912-019-0846-7

