# Supplementary material for: Asparagine endopeptidase regulates lysosome homeostasis via modulating endomembrane phosphoinositide composition
Source: Cell Death Dis. 2025 Jan 2;15(12):883. doi: 10.1038/s41419-024-07187-3 (PMC11693757; doi:10.1038/s41419-024-07187-3)
Supplement: Supplementary file 1 — Supplemental figures [file 41419_2024_7187_MOESM1_ESM.docx]

**Supplementary Figures**

**Asparagine endopeptidase regulates lysosome homeostasis via modulating endomembrane phosphoinositide composition**

**Linli Yao**^2^†**, GuangHui Zi**^1^†**,** Miao He^1^, Yuhong Xu^1,3^, Lulu Wang^5^, **Baowei Peng**^1,3,4^*****

^1^College of Pharmacy, Dali University, Dali 671003, Yunnan Province, P.R. China

^2^State Key Laboratory of Oncogenes and Related Genes, Shanghai Cancer Institute, Ren Ji Hospital, School of Medicine, Shanghai Jiao Tong University, Shanghai, P.R. China

^3^Yunnan Key Laboratory of Screening and Research on Anti-pathogenic Plant Resources from Western Yunnan. Dali University, Yunnan Province, P.R. China

^4^Yunnan Provincial Key Laboratory of Entomological Biopharmaceutical R&D, College of Pharmacy, Dali University, Dali, Yunnan, P.R. China

^5^Department of Human Anatomy, School of Basic Medical Sciences, Capital Medical University, Beijing, P.R. China

**†These authors contributed equally to this work.**

***Corresponding authors:**

Baowei Peng, College of Pharmacy, Dali University, Dali 671003, Yunnan Province, P.R. China. E-mail: pengbaowei@dali.edu.cn.

**Short Title**：AEP promotes breast cancer progression.


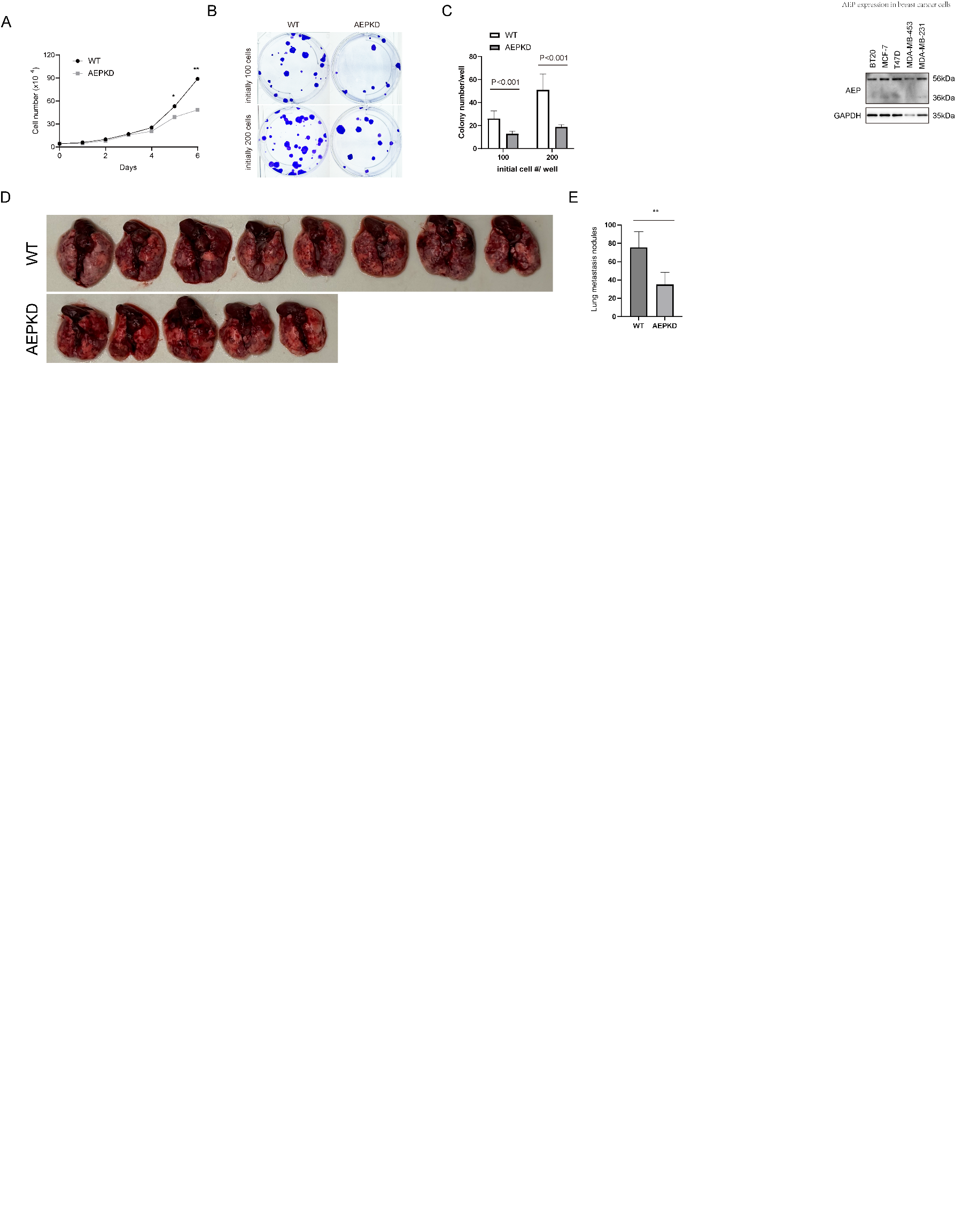


**Supplementary Fig. 1. AEP knockdown in 4T1 cells suppressed cell survival and lung metastasis.** **A**, AEP knockdown in 4T1 cells suppresses cell growth. **B** and **C**, AEP knockdown in 4T1 cells impairs colony formation *in vitro*. The image shown is from representative experiments The right graphs showing quantification of colony number/well and colony size. Data are mean ± s.d.; n=3. Similar results are observed in four independent experiments. **D**, Loss of AEP in 4T1 cells suppressed cancer cell metastasis *in vivo*. WT and AEPKD 4T1 cells were inoculated into nude mice via tail vein injection. Mice lungs were dissected and gross morphology of lung was observed. **E,** Quantification of lung metastatic nodules in mice. Data are mean ± s.d.; n=8 mice for WT group and n=5 mice for AEPKD group.


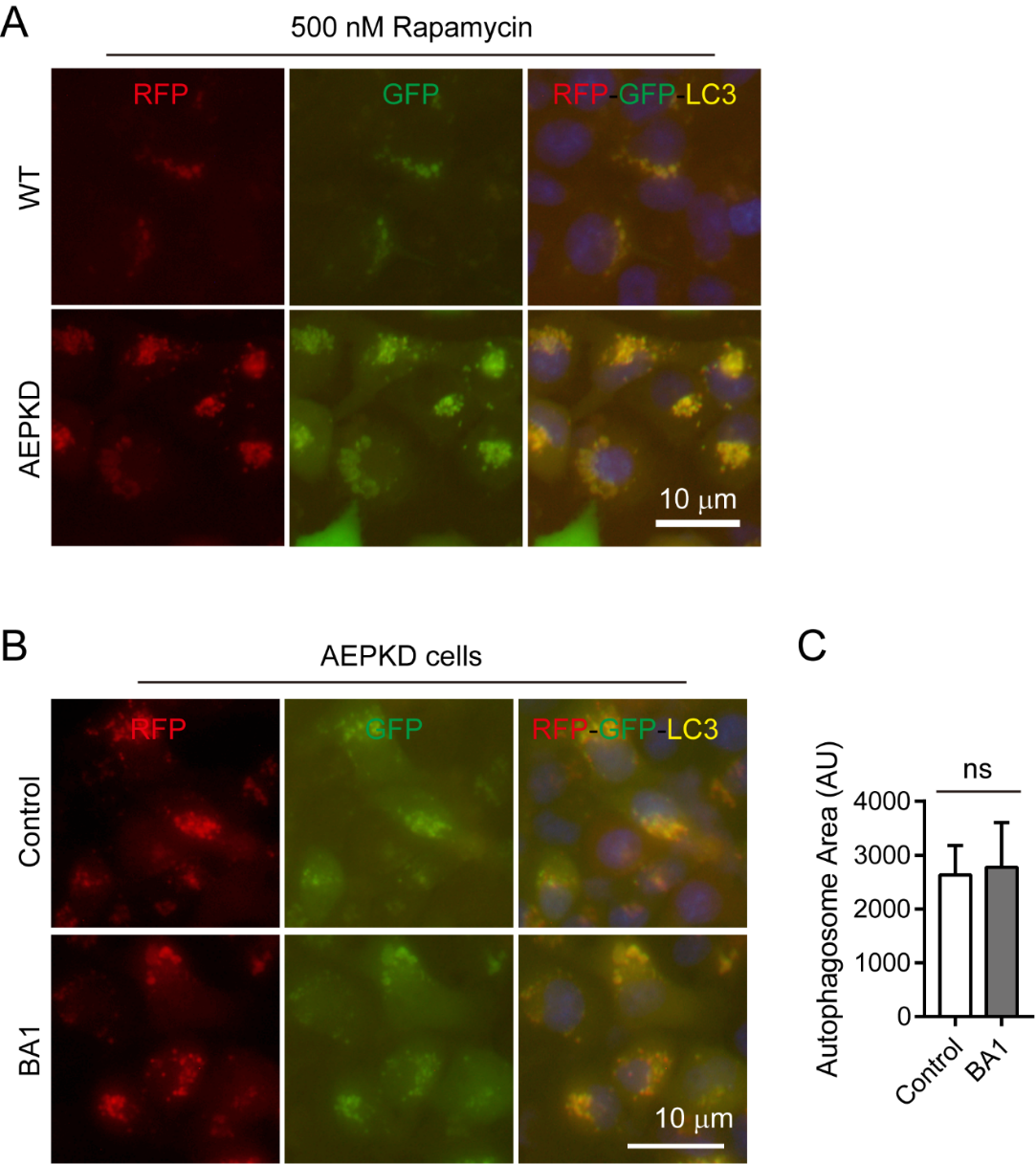
**Supplementary Fig. 2. Effect of inducing autophagosome synthesis and inhibiting lysosome acidification on autophagosome accumulation in serum-starved AEPKD cells. A**, MDA-MB-231 cells expressing tandem mRFP-GFP-LC3 in full tissue-culture medium were treated with 500 nM Rapamycin for 16 h and examined by fluorescence microscopy. Rapamycin induced greater autophagosome accumulation in AEPKD cells than in WT cells. **B**, BA1 did not significantly increase autophagosome accumulation in serum-starved AEPKD cells. AEPKD cells expressing tandem mRFP-GFP-LC3 were deprived of FBS for 16 h in the presence or absence of 100 nM BA1 and examined by fluorescence microscopy. **C**, Quantification of autophagosome area in **B**. Data are mean ± s.d.; n=50 cells from representative experiments. n.s. not significant by two-tailed t-test. Results shown are from representative experiments.


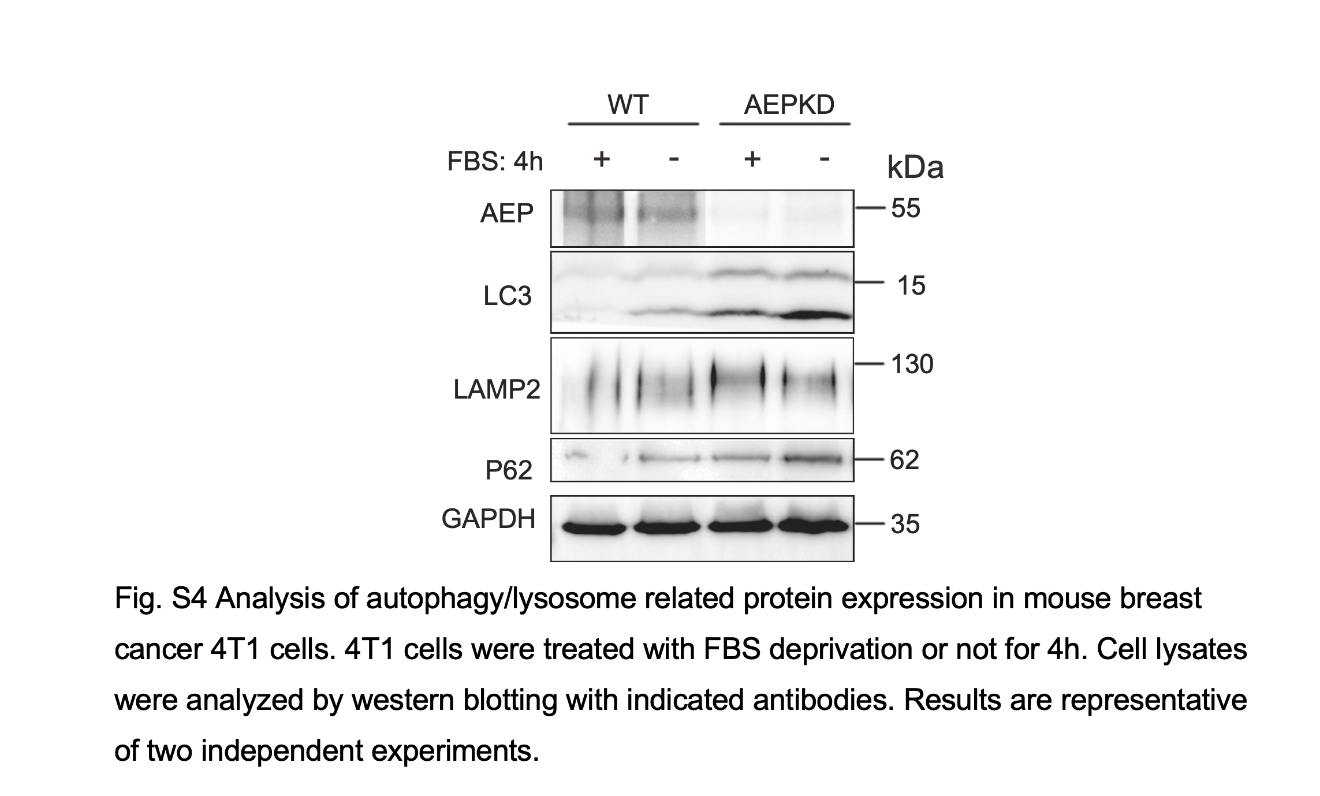


**Supplementary Fig. 3**. **Analysis of autophagy/lysosome related protein expression in mouse breast cancer 4T1 cells.** 4T1 cells were treated with FBS deprivation or not for 4h. Cell lysates were analyzed by western blotting with indicated antibodies. Results are representative of two independent experiments.


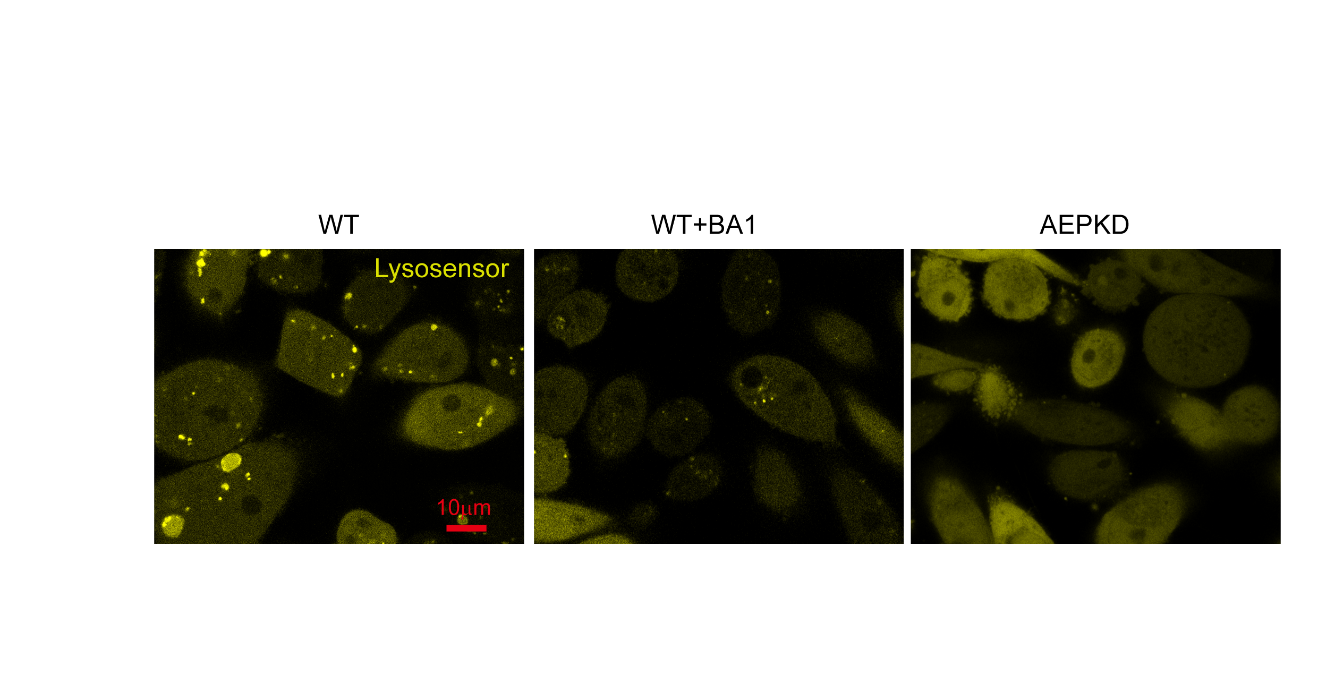


**Supplementary Fig. 4. Effect of BA1 treatment on Lysosensor labeling of lysosomes.** Confocal microscopy observation of cells labeled with Lysosensor showing it concentrated in lysosomes in WT cells but not in WT treated with BA1 or AEPKD cells. MDA-MB-231 WT cells, WT cells treated with 100 nM BA1 overnight, and AEPKD were labeled with 2.5 µM lysosensor for 10 min and imaged live. The image shown is from representative experiments.


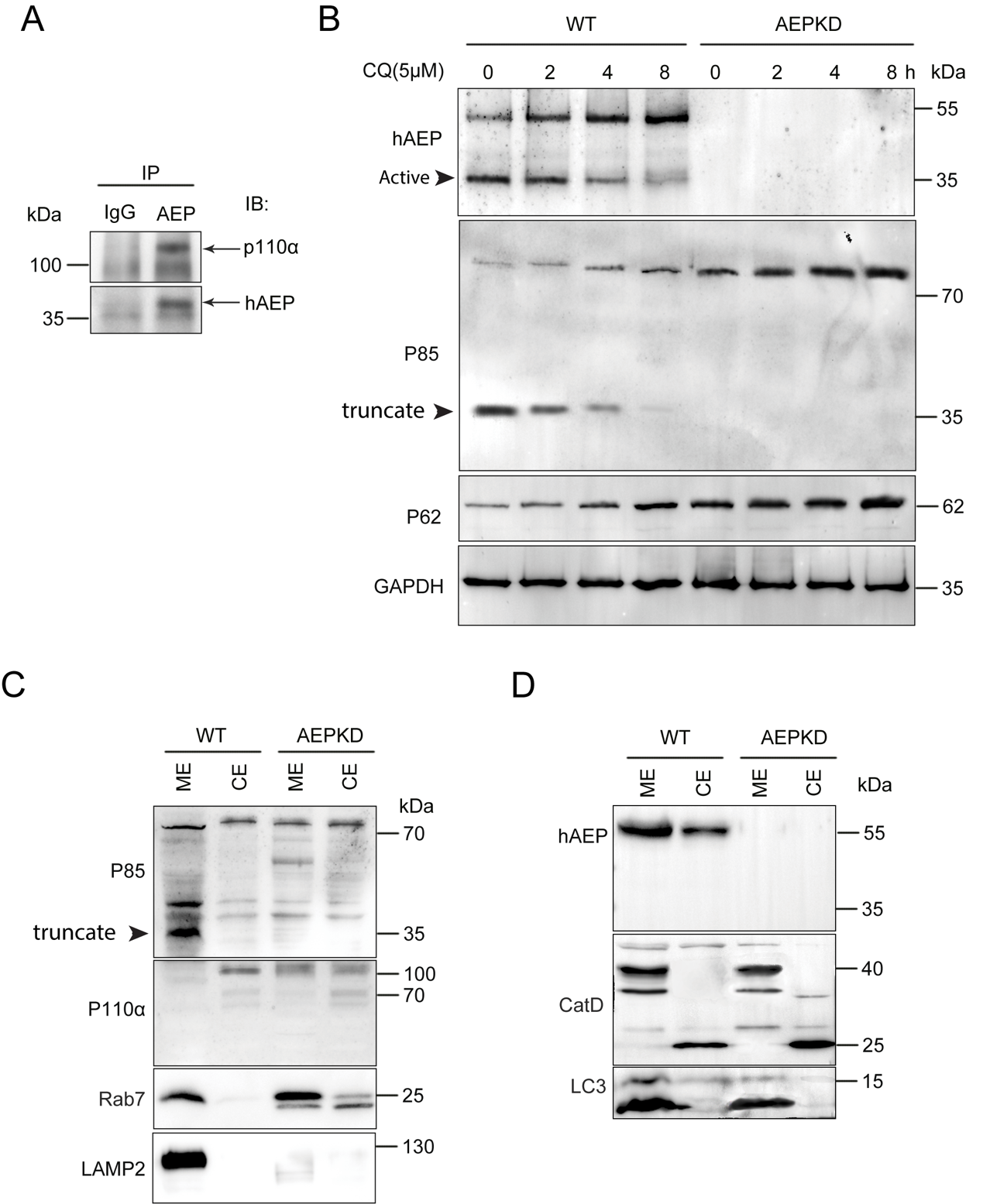


**Supplementary Fig. 5. Analysis of interaction between p85 and AEP in mouse breast cancer 4T1 cells.** A, Co-IP of p110a by AEP. B, Cleavage of p85 correlated with AEP activation, which was regulated by CQ. C, D, Cells were fractionated into ME and CE, which were analyzed by WB to examine distribution of indicated proteins.


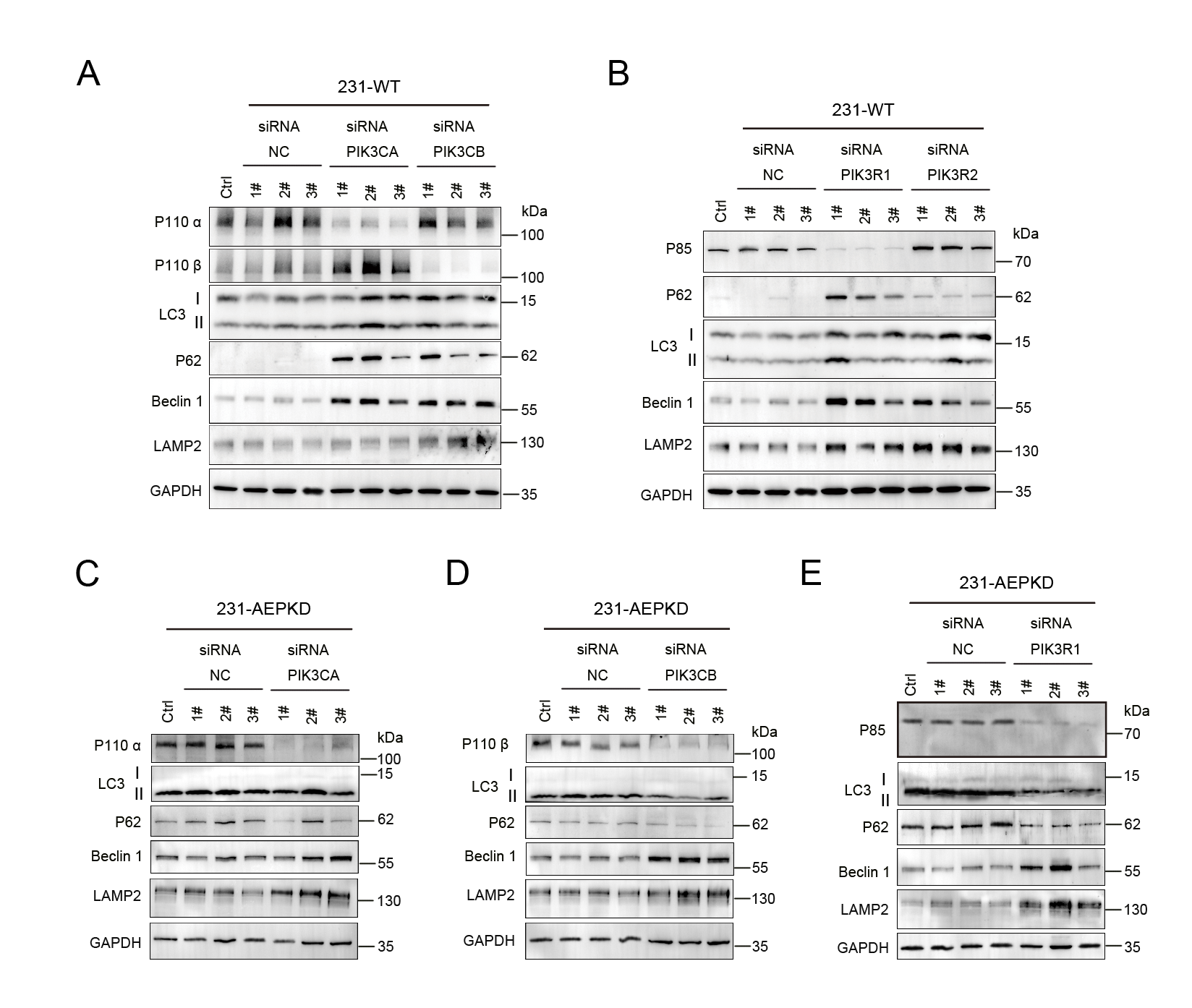


**Supplementary Fig. 6. Analysis of the effect of PI3K in AEP WT and AEPKD MDA-MB-231 cells.** A, B, 231-WT cells were treated with siRNAs specific for the catalytic subunits (PIK3CA, PIK3CB) or regulatory subunits (PIK3R1, PIK3R2). Three different siRNAs for each target were used. Cells were harvested 36 h later, lysed and analyzed for the expression of proteins with indicated antibodies. C, D, E, 231-AEPKD cells were treated with siRNAs specific for catalytic subunit PIK3CA (C), catalytic subunit PIK3CB (D), and regulatory subunit PIK3R1 (E). Three different siRNAs for each target were used. Cells were harvested 36 h later, lysed and analyzed for the expression of proteins with indicated antibodies.


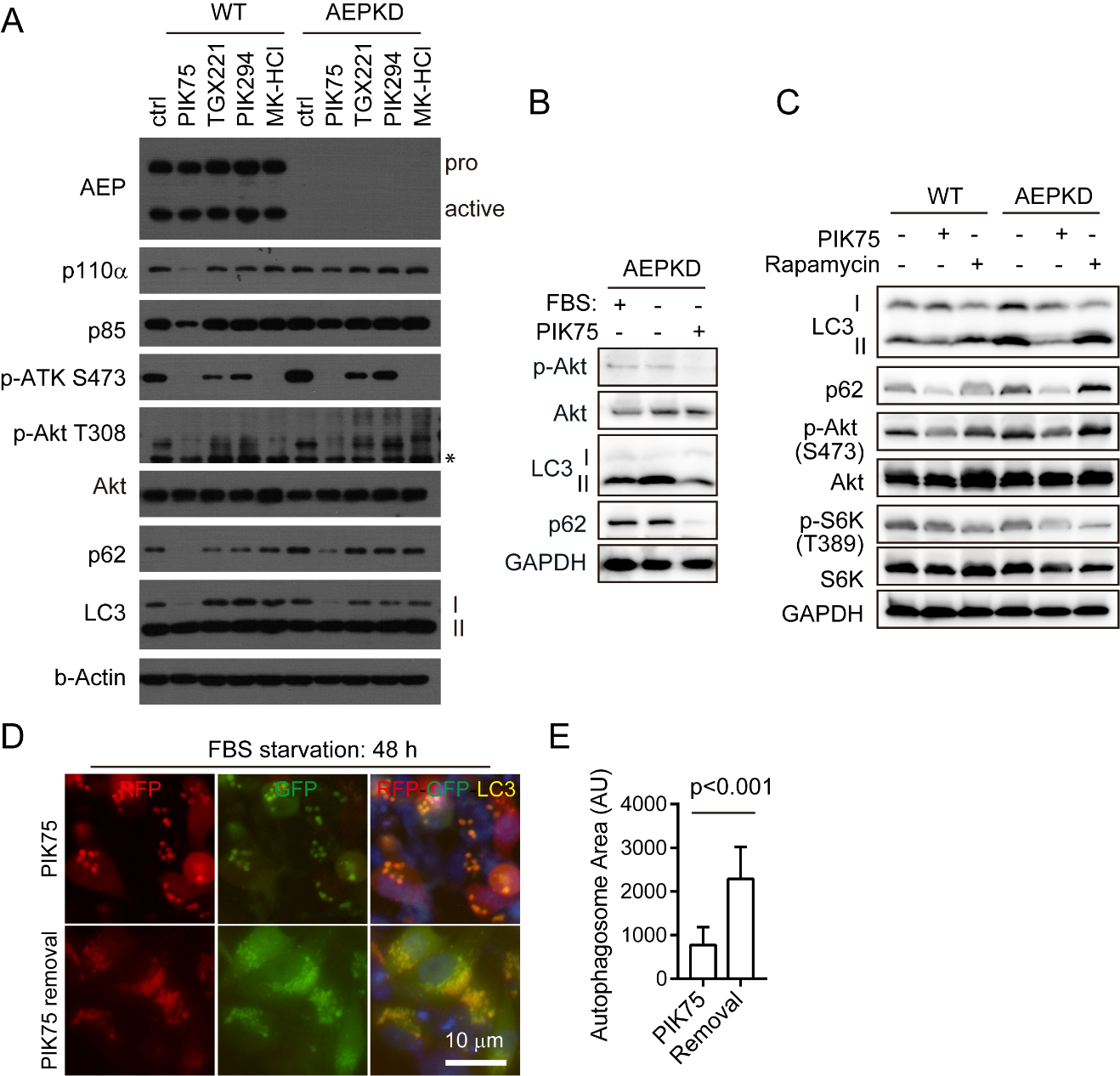


**Supplementary Fig. 7. PIK75 is a potent PI3K inhibitor. A**, PIK75 induces autophagy clearance of p62. WT and AEPKD MDA-MB-231 cells were treated with PI3K inhibitors PIK75 (100 nM), TGX221 (200 nM), PIK294 (200 nM) and Akt inhibitor MK-HCl (200 nM) overnight. Cell lysates were analyzed by immunoblotting with antibodies specific to AEP, PI3K (p85 & p110a), Akt, phosphorylated Akt (S473 & T308), p62 and LC3. PIK75 inhibited PI3K activity and induced autophagy clearance. PIK75 treatment also led to degradation of p85 and p110a, especially in WT cells. *, non-specific band. The immunoblot shown is from representative experiments. **B**, PIK75 promotes autophagy clearance in serum-starved cells. MDA-MB-231 cells were starved for FBS in the presence or absence of PIK75 for the last 5 h. Cell lysates were analyzed by immunoblotting with antibodies as indicated. The immunoblot shown is from representative experiments. **C**, Comparison of PIK75 and Rapamycin on autophagy and autophagic clearance. MDA-MB-231 cells were treated with 100 nM PIK75 or 500 nM Rapamycin overnight. Cell lysates were analyzed by immunoblotting with antibodies as indicated. PIK75, but not Rapamycin, promoted clearance of autophagic cargo. The immunoblot shown is from representative experiments. **D**, Removal of PIK75 resulted in re-accumulation of autophagosomes. AEPKD MDA-MB-231 cells expressing mRFP-GFP-tfLC3 were deprived of FBS for 24 h, in the presence or absence of 100 nM PIK75 for the last 5 h, which was removed by washing with cold PBS. Cells were cultured for another 24 h in FBS deprived culture medium and imaged live to examine autophagosomes. The image shown is from representative experiments. **E**, Quantification of autophagosome area in **J**. Data are mean ± s.d.; n=50 cells from representative experiments. p<0.001 by two-tailed t-test.
